# An atypical class of non-coding small RNAs is produced in rice leaves upon bacterial infection

**DOI:** 10.1038/s41598-021-03391-9

**Published:** 2021-12-17

**Authors:** Ganna Reshetnyak, Jonathan M. Jacobs, Florence Auguy, Coline Sciallano, Lisa Claude, Clemence Medina, Alvaro L. Perez-Quintero, Aurore Comte, Emilie Thomas, Adam Bogdanove, Ralf Koebnik, Boris Szurek, Anne Dievart, Christophe Brugidou, Severine Lacombe, Sebastien Cunnac

**Affiliations:** 1 PHIM Plant Health Institute, Univ Montpellier, IRD, CIRAD, INRAE, Institut Agro, Montpellier, France; 2 Plant Pathology and Plant-Microbe Biology Section, School of Integrative Plant Science, Cornell University, Ithaca, NY USA; 3 UMR AGAP Institut, Univ Montpellier, CIRAD, INRAE, Institut Agro, 34398 Montpellier, France; 4 CIRAD, UMR AGAP Institut, 34398 Montpellier, France; 5 Present Address: Department of Plant Pathology, The Ohio State University, Columbus, OH 43201 USA; 6 Present Address: Infectious Disease Institute, The Ohio State University, Columbus, OH 43201 USA

**Keywords:** Plant immunity, Small RNAs, Plant molecular biology

## Abstract

Non-coding small RNAs (sRNA) act as mediators of gene silencing and regulate plant growth, development and stress responses. Early insights into plant sRNAs established a role in antiviral defense and they are now extensively studied across plant–microbe interactions. Here, sRNA sequencing discovered a class of sRNA in rice (*Oryza sativa*) specifically associated with foliar diseases caused by *Xanthomonas oryzae* bacteria. *Xanthomonas*-induced small RNAs (xisRNAs) loci were distinctively upregulated in response to diverse virulent strains at an early stage of infection producing a single duplex of 20–22 nt sRNAs. xisRNAs production was dependent on the Type III secretion system*,* a major bacterial virulence factor for host colonization. xisRNA loci overlap with annotated transcripts sequences, with about half of them encoding protein kinase domain proteins. A number of the corresponding rice *cis-*genes have documented functions in immune signaling and xisRNA loci predominantly coincide with the coding sequence of a conserved kinase motif. xisRNAs exhibit features of small interfering RNAs and their biosynthesis depend on canonical components *OsDCL1* and *OsHEN1.* xisRNA induction possibly mediates post-transcriptional gene silencing but they do not broadly suppress *cis*-genes expression on the basis of mRNA-seq data. Overall, our results identify a group of unusual sRNAs with a potential role in plant–microbe interactions.

## Introduction

RNA silencing represents a fundamental mechanism of gene regulation in eukaryotes and orchestrates a wide range of basic cellular and organismal processes. In plants, RNA silencing coordinates major biological functions such as maintenance of genome integrity and inheritance, development and environmental responses^1, 2^. Non-coding small RNAs (sRNAs) determine the specificity of RNA silencing. sRNAs are typically 20–24 nucleotides (nt) in size and derive from a double stranded RNA (dsRNA) precursor that is diced into a single or multiple distinct sRNA duplexes by Dicer-like proteins (DCL). In plants, the released sRNAs duplexes are universally methylated on the 2′-OH of the 3′ terminal ribose of each strand by HEN1^3^. The mature sRNA duplex ‘guide’ strand is selectively loaded into an ARGONAUTE (AGO) protein component of RNA-Induced gene Silencing Complexes to mediate either transcriptional gene silencing (TGS) or post-transcriptional gene silencing (PTGS) according to nucleic acids sequence complementarity between the sRNAs and their target RNA^4^. TGS typically depends on 24 nt heterochromatic small interfering RNAs (het-siRNAs) and involves DNA methylation and chromatin reprogramming. PTGS, on the other hand, guided by 21 nt micro and siRNAs, leads to mRNA cleavage and mRNA translational inhibition^1^.

Several major classes of endogenous plant regulatory sRNAs have been defined based on the specifics of their biogenesis pathways and their mode of action^3, 5^: Micro RNAs (miRNAs) are arguably the best characterized class of sRNAs. Hairpins of *MIRNA* gene transcripts (pri-miRNA) are cleaved and diced by the plant DCL1 protein to generate a single miRNA duplex. Other hairpin small interfering RNAs (hp-siRNAs) also derive from a single-stranded RNA with inverted repeats that folds into a hairpin structure but whose processing does not follow the precise pattern associated with miRNAs. The remaining main classes of sRNAs pertain to siRNAs whose dsRNA precursor is made up of two complementary RNA molecules. With the exception of Natural antisense transcript (NAT) siRNAs (NAT-siRNAs) that are produced from two independently transcribed but complementary precursors, siRNAs biogenesis usually involves an RNA-dependent RNA polymerase (RDR) catalyzed reaction. For example, phased secondary siRNAs (phasiRNAs) and *trans*-acting siRNAs are generated following the conversion of a single-stranded miRNA cleavage product to a double stranded precursor by RDR6^6^.

Even if the role of sRNAs in plant biotic interactions is an area of intensive research, it is well-established that multiple plant RNA silencing pathways are also critical for plant immune responses to bacterial, fungal or oomycete pathogens^7^. In nature, healthy plants rely on their innate immune system to perceive and fight off pathogens. PAMP-triggered immunity (PTI) represents a first line of defense relying on the detection of Pathogen-Associated Molecular Patterns (PAMPs) such as bacterial flagellin or fungal chitin. Plasma membrane localized receptor-kinases and receptor-like proteins perceive PAMPs and trigger phosphorylation cascades that ultimately signal the deployment of defense mechanisms to hinder pathogen invasion^8^. Plant receptor-kinases (a.k.a. RLKs for Receptor-Like Kinases) have a monophyletic origin and have been classified into the RLK/Pelle kinase family^9, 10^. This family also includes Receptor-Like Cytoplasmic Kinases (RLCKs) that lack the transmembrane and extracellular domains and function intracellularly as substrates of receptor-kinase complexes for signal transduction^11^.

Several plant miRNAs whose expression is modulated in response to PAMPs were shown to be important to efficiently combat pathogen attacks downstream of the perception of PAMP signals^7^. Adapted pathogens have evolved mechanisms to counteract eukaryotic immune responses. For instance, many Gram-negative bacterial pathogens employ a Type Three Secretion System (T3SS) to inject effector proteins directly into eukaryotic host cells to modulate their physiology and suppress defenses^12^. Similar to Viral Suppressors of RNA silencing plant-pathogenic bacteria and oomycetes have been shown to use their repertoire of virulence effector proteins to dampen sRNA silencing activity and compromise plant immunity^13^. As a counter-defense mechanism, plants evolved resistance (R) proteins that recognize pathogen virulence factors through a mechanism called Effector-Triggered Immunity (ETI) which restricts pathogen growth and infection by triggering programmed cell death^14^. The role of sRNA as positive mediators of immunity is not limited to the PTI layer, and both TGS and PTGS also regulate the ETI layer^7^.

Rice (*Oryza sativa*) is a major crop in global agriculture and stands as an economic and nutritional cornerstone in the developing world. Rice possesses RNA silencing pathways that are both analogous to *A. thaliana* and idiosyncratic^15, 16^. *Xanthomonas oryzae* pv. *oryzae* (*Xoo*) and *X. oryzae* pv. *oryzicola* (*Xoc*) are the pathogenic agents that cause major diseases of rice: bacterial leaf blight (BLB) and bacterial leaf streak (BLS), respectively. Even though *Xoo* and *Xoc* belong to the same *X. oryzae* (*Xo*) species, they form distinct phylogenetic groups that are further delimited as a function of their geographic origin and have contrasted infection strategies*: Xoo* enters through hydathodes and invades the xylem whereas *Xoc* uses stomata to colonize the plant mesophyll^17^. *Xo* strains employ virulence factors, including the T3SS, that contribute to disease development. Mutants lacking the T3SS are unable to translocate effector proteins into host cells and are non-virulent. These bacteria possess over 32 effectors, including a unique class of Transcription Activator-Like Effector (TALE). TALEs activate protein-encoding gene expression and reprogram the transcriptome of the host’s cells by mimicking eukaryotic transcription factors^12^.

Recently, the discovery of trans-kingdom RNAi or *trans*-species small RNA silencing, has shed a new light on the role of sRNAs as mobile effectors modulating the outcome of plant biotic interactions^7, 18^. Pathogen effectors acting as Suppressors of RNA silencing and pathogen *trans*-species sRNAs are both delivered into the plant cell to prevent defense. However, while the former proteinaceous effectors suppress silencing, the latter ribonucleic effectors instead, exploit it. There is still limited evidence in support of a third virulence strategy where proteinaceous effectors would leverage RNA silencing to promote immune suppression and susceptibility. Recently however, a *Xoo* T3SS effector-induced rice miRNA was shown to exert PTGS on a *R* gene conferring quantitative resistance to BLB^19^.

With a few exceptions^7^, the available functional data on the role of endogenous plant sRNAs in regulating the disease process has focused almost exclusively on miRNAs and their role in promoting immunity. For example, in rice, the expression of multiple miRNAs changes in response to the blast fungus *Magnaporthe oryzae*^20^ and *Xoo*^21, 22^. Here, we aimed at examining all sRNAs associated with the disease process at a genome-wide scale and used an unbiased small RNA deep-sequencing data analysis approach on rice inoculated with *Xo* strains. Unusual 20–22 nt small RNAs were found to be generated from annotated protein coding loci in the *Xo* infected leaf in a T3SS-dependant manner and required the archetypal sRNA biogenesis pathways components *OsDCL1* and *OsHEN1*. Several xisRNAs mapped to *cis*-genes previously implicated in immune signaling, but did not appear to generally operate through PTGS of their *cis*-gene. This study provides insight into an intriguing group of sRNAs that may participate in gene silencing for plant disease development.

## Results

### Small RNA sequencing of rice leaves infected with Xoo BAI3 identifies sRNA loci that are upregulated in a T3SS-dependant manner

To gain genome-wide insight on host sRNA species that may be involved in the disease process, we generated sRNA Illumina sequencing data from RNA collected from the Japonica *O. sativa* rice cultivar Nipponbare leaves confronted by pathogenic Xoo. Briefly, sRNAs were analyzed from leaf samples taken 24 h post-infiltration (hpi) with the wild-type African Xoo reference strain BAI3, a non-virulent T3SS mutant, which is unable to inject virulence effectors (herein referred to as BAI3H) or a water control (H2O). We inferred potential sRNA generating loci based on sRNA reads coverage profile along the Nipponbare genomic sequences, and tested for differential accumulation of sRNA reads in treatment comparisons as summarized in Supplementary Fig. S1. Because the T3SS is an essential component of infection, we aimed to define sRNA loci that were induced in a T3SS-dependent fashion. We required that Xanthomonas-induced small RNA (xisRNA) loci be significantly induced in both the BAI3 vs H2O and the BAI3 vs BAI3H comparisons but not in the BAI3H vs H20 comparison where no T3SS effect is expected to occur. To ensure that xisRNA loci would not correspond to repetitive sequences and would be enriched for potential regulatory non-coding sRNAs, we further required that ≥ 75% of the reads at these loci mapped uniquely in the genome and that ≥ 50% of the reads had a size between 20 and 24 nt. Applying these criteria to the set of 341 Differentially Expressed (DE) sRNAs loci resulted in a total of 64 xisRNA loci.

**Figure 1:**
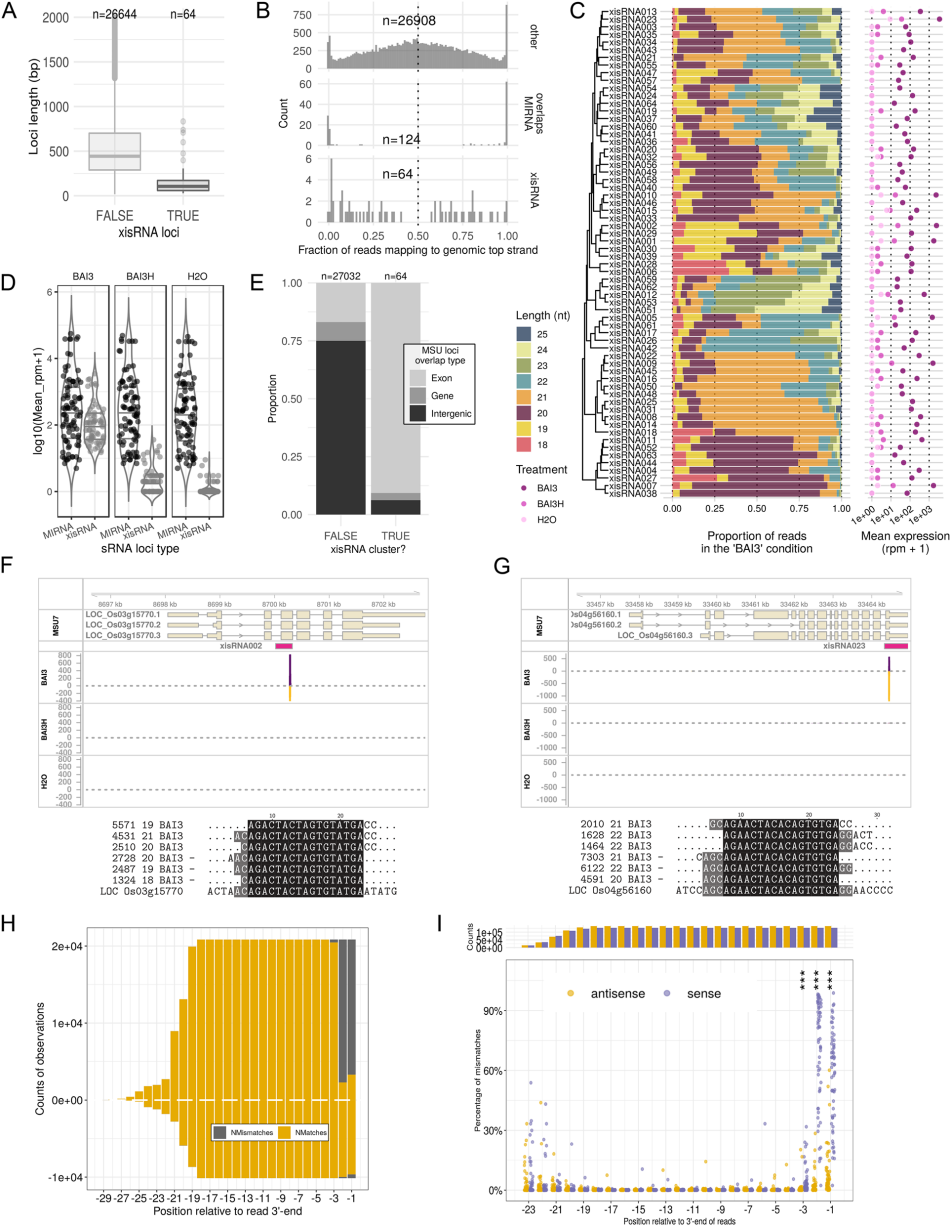
Xanthomonas-induced small RNAs (xisRNA) signatures and loci possess distinctive attributes. (**A**) Box plot representation (median, 1st and 3rd quartile and 1.5 × IQR) of the distribution of the length of the experimental sRNA loci, contrasted as a function of whether they correspond to a xisRNA or not. Note that 388 loci were excluded from the plot because their length is above 2000 bp. (**B**) Distribution of the fraction of reads mapping to the genomic top strand for experimental sRNA loci contrasted as a function of whether the loci correspond to xisRNA, MIRNA or other loci. (**C**) Distribution of sRNA reads length and mean expression at the 64 xisRNA loci. The hierarchical clustering of xisRNA loci is based on the profiles of reads proportions for individual length values. Mean expression values in each treatment (shades of pink) are represented on a log10 scale. (**D**) Distribution of mean normalized expression in reads per million (rpm) for pri-miRNA loci (n = 87) versus xisRNA loci (n = 64) in libraries derived from various leaf inoculation treatments. (**E**) Proportions of the type of rice annotation features overlapping with experimental sRNA loci, contrasted as a function of whether sRNA loci correspond to a xisRNA or not. Overlaps are included in the counts if spanning at least 20 bp. Experimental sRNA loci overlapping both a gene and an exon annotation range are classified as ‘Exon’. Those overlapping a gene annotation element only are classified as ‘Gene’. Others are classified as ‘Intergenic’. (**F**) Genome browser snapshot of the MSU7 LOC_Os03g15770 locus annotation overlapping with the xisRNA002 ShortStack segment and read coverage tracks (reads per million) for the various experimental treatments. Shorter exon boxes correspond to untranslated regions. Coverage of reads mapping to the top genomic strand is represented with positive values and a violet colored area whereas coverage of reads mapping to the opposite strand is represented with negative values and a golden colored area. The multiple sequence alignment below the genome browser graph corresponds to the three most abundant unique mapping signatures per strand of the ShortStack loci as well as the genomic sequence (LOC ID). In the sequence title, the two numbers indicate, respectively, the count of reads with this sequence in the “BAI3” treatment libraries and the length of the unique sequence. The “–” sign indicates that the original read sequences map to the complementary strand of the genomic locus. In this case, the displayed sequences are thus the reverse complement of the original read sequences. (**G**) Same as E but for the LOC_Os04g56160 locus region overlapping with xisRNA023. (**H**) Counts of nucleotides matching (Nmatches) or not matching (Nmismatches) the xisRNA002 loci genomic sequences along the read alignment region. Positions on the x-axis are expressed relative to the sRNA reads 3′-end (last nucleotide is − 1). Positive values are computed for reads mapping to the annotated sense strand of the LOC_Os03g15770 locus. Conversely, negative values correspond to reads mapping to the antisense strand of the annotated loci. (**I**) Distribution of genomic sequence mismatch ratios along relative positions within reads for xisRNA loci. Each data point represents the ratio of the number of mismatches over the total number of comparisons with the genomic sequence of the MSU annotated transcript for that position of all the reads mapping to a xisRNA locus in the sense orientation (violet) or antisense orientation (golden). Positions where a two-sided Wilcoxon signed rank test measuring differences in xisRNA mismatch ratios between read orientations were significant (BY adjusted p-value ≤ 0.05) are marked with a “*” sign. Here, three “*” indicate an adj. P-val. ≤ 0.001. The bar graph on the top reports on the total count of comparisons for each orientation.

To characterize xisRNA loci, we first analyzed the distribution of xisRNA loci length (Fig. 1A) and found that with a median of 106 base pairs (bp), their length is significantly different from other experimentally defined sRNA loci (Two-sided Wilcoxon rank sum test: W = 1,547,014, p = 1.02e−27). In addition, inspecting the distribution of the proportion of reads mapping to the top genomic strand (Fig. 1B), revealed that, as expected, a vast majority of the experimental sRNA loci overlapping pri-miRNA genomic intervals have mapping strand ratios of 0 or 1. In contrast, the bulk of xisRNA and other experimental sRNA loci are composed of reads partitions that map to either strands of their cognate genomic locus, reminiscent of siRNA loci. xisRNA loci expression in non-inducing conditions is extremely low or even null (Fig. 1C,D). In contrast, upon BAI3 infection, xisRNA loci reads are rather abundant. Even if overall lower, their normalized expression is not at odd with pri-miRNA loci levels (Fig. 1D). The most highly expressed loci, namely, xisRNA023, 01, 02, 05, 07, 09 and 10 reach several thousand rpm in leaves inoculated with virulent BAI3 bacteria (Fig. 1C). Regarding read sizes distributions, no major global bias in *MIRNA* genes-derived reads sizes and coverage patterns were detected (Supplementary Fig. S2). While this suggests that read populations in our dataset are not anomalously biased, read length distribution for xisRNA loci was broader than for *MIRNA* genes (Fig. 1C). Furthermore, with a few exceptions (e.g. xiRNA059, 62, 12, 53 and 51), xisRNA read sizes ranged overall from 20 to 22 nt, which is comparable to rice siRNAs.

In order to relate xisRNA loci with existing rice miRNA annotation, we asked whether they overlap with *O. sativa MIRNA* gene loci annotated in MirBase but found no match. Consistent with this observation, none of the xisRNA loci was classified by ShortStack^23^ as a possible MIRNA locus (category ‘N14’, ‘N15’ or ‘Y’), suggesting that their features are incompatible with their being previously undocumented MIRNA genes. We also examined the overlap of experimental sRNA loci with annotated genes and transcripts in the Rice Genome Annotation Project (MSU7 annotation)^24^. As illustrated in Fig. 1E, the distribution of the overlap type with MSU loci is different for xisRNA loci as compared to other experimental sRNA loci. Notably, 58 out of 64 xisRNA loci (91%) overlap with an annotated MSU exon which is significantly greater than for other experimental sRNA loci (One-sided Fisher's Exact test adjusted p-value = 1.57e−37).

In summary, xisRNA loci are generally short and do not correspond to documented or putative *MIRNA* genes but a majority of them overlap with exons of annotated rice genes. Analogous to siRNAs, these loci are composed of 20–22 nucleotides-long reads mapping to both strands of the genomic sequence and that can also accumulate to substantial levels in the context of a T3SS-dependent *Xoo* BAI3 infection.

### BAI3 xisRNAs loci generate single sRNA duplexes

A majority of xisRNA loci overlap with exons of annotated protein-coding genes. We therefore examined the coverage profile of mapped xisRNAs signatures along the Nipponbare genome sequence in regions encompassing annotated MSU loci (see individual coverage plots in our dataverse). For example, highly expressed and typical xisRNA002 and xisRNA023 loci display a T3SS-dependant ~ 25 bp-wide coverage peak comprised of reads mapping on an annotated exon in either sense or antisense orientation (Fig. 1F,G). To extent this observation, we inspected read mappings at these genomic locations (Supplementary Fig. S3) and created multiple alignments of the most abundant unique read signatures for each strand of the xisRNA loci (Fig. 1F,G). On these alignments, the pattern of complementarity between sense and anti-sense xisRNA major reads, with ~ 2 nt 3′-overhangs, is evocative of guide-passenger strands of sRNAs duplexes resulting from DCL processing. Another discernible feature is that the last two 3′-end nucleotides of major reads in the sense orientation relative to the annotated *cis*-transcript do not match the transcript sequence. To evaluate whether the presence of untemplated 3′-most nucleotides is a general feature of xisRNA sense reads, we computed the counts of matches and mismatches relative to the genomic sequence for individual positions along read sequences in each xisRNA loci and contrasted them as a function of read orientation (exemplified for xisRNA002 in Fig. 1H). This further enabled to derive the position- and strand-specific mismatch ratios distributions plotted in Fig. 1I for all xisRNA loci. Statistical tests for differences in xisRNA mismatch ratios for individual positions were significant (adjusted p-value ≤ 0.05) only for the last first, second and third nucleotides (positions − 1, − 2 and − 3) where xisRNA loci displayed overall higher proportions of mismatches for sense reads than for antisense reads. The substitution matrix of genomic sequence versus reads sequences for positions − 2 and − 1 in Supplementary Fig. S4 indicates that xisRNA sense reads untemplated 3′ nucleotides are overwhelmingly biased for cytosines, independently of the nature of the underlying genomic nucleotide. To rule out the possibility that this predominance of 3′ untemplated nucleotides in sense xsiRNA reads would be a global artifact of our Illumina sequencing experiment, we conducted the same analysis for a subset of non-xisRNA experimental sRNA loci and did not conclude on any significant differences in mismatch ratios (Supplementary Fig. S4). In contrast, testing differences in sense reads mismatch ratios between pri-miRNA transcripts and xisRNA loci indicated that these ratios were significantly higher for xisRNA than for pri-miRNA loci (Supplementary Fig. S4).

In short, we obtained evidence that while similar to non-coding small RNAs loci, xisRNA loci are essentially composed of complementary reads reminiscent of a single sRNA duplex with those reads in the sense orientation relative to an annotated *cis*-mRNA having a high proportion of untemplated cytosines in the last 2–3 3′ nucleotides.

### *X. oryzae* strains from multiple sublineages induce xisRNAs

**Figure 2:**
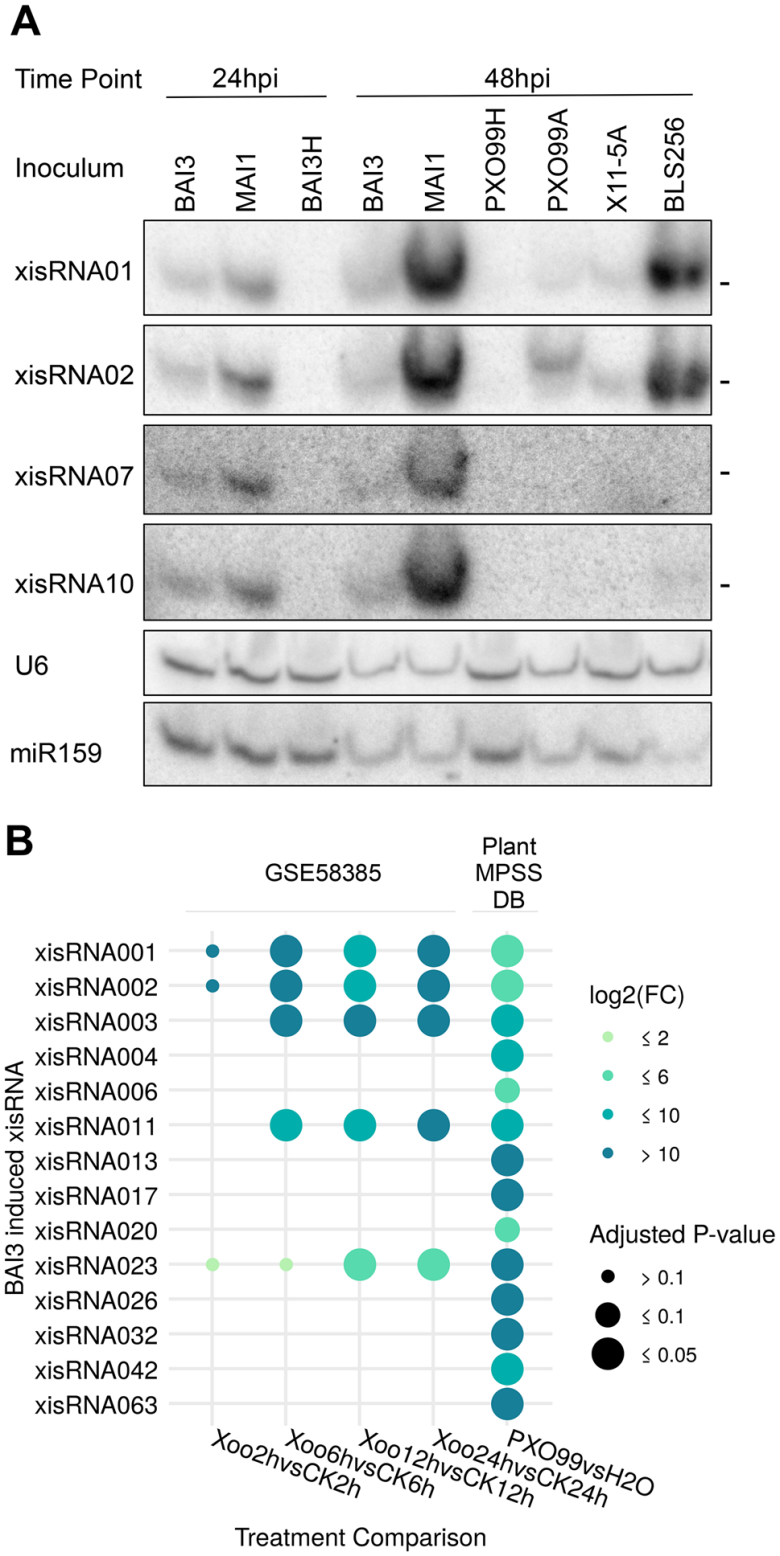
A subset of xisRNAs are detected in northern blots and exhibit contrasted expression patterns in response to phylogenetically diverse Xo strains. (**A**) Autoradiographs obtained after RNA gel northern blot analysis conducted with specific oligonucleotide probes hybridized to total RNA extracted from Nipponbare leaves infiltrated with the indicated strains and collected at the indicated time points (*hpi* hours post inoculation). The xisRNA probes detect RNAs complementary to the sense strand of the xisRNA *cis*-gene. The BAI3H and PXO99H strains correspond to a T3SS defective mutant derivative of the BAI3 and PXO99A virulent strains, respectively. The hyphens on the right of individual blots mark a size of 21 nt. Individual images derive from successive stripping, hybridization and detection rounds of the same membrane. Full-length blots are presented in Supplementary Fig. S15. Equivalent results were obtained in two other similar experiments. (**B**) Differential expression statistics of xisRNA loci found to be induced by the Asian Xoo PXO99 strain in public sRNA-seq datasets. The treatment comparisons (bottom labels) involve tissue samples inoculated with PXO99 versus a mock control at the indicated time point. The plot displays xisRNA loci with a log2(fold change) ≥ 1 and an BH adjusted p-values ≤ 0.1 in at least one comparison.

In order to independently validate the assumption that xisRNA NGS reads correspond to genuine sRNA species, we performed standard low molecular weight northern blots with probes designed to detect the moderately to highly expressed xisRNA duplex strands that are complementary to annotated *cis*-transcripts. In agreement with this view and as shown in Fig. 2A and Supplementary Fig. S5, for all tested xisRNA loci, following Nipponbare leaf infiltration with the BAI3 strain, a T3SS-dependent signal was detected no earlier that 24 hpi and gradually increased in intensity up to 72 hpi. We were curious to determine if the capacity to trigger xisRNA expression was specific to *Xoo* BAI3 alone or if it is a conserved feature of the phylogenetically diverse *Xo* species. Northern blot experiments were conducted with RNA samples extracted from leaves infiltrated with reference strains representative of the main genetic and pathotypic groups: African (BAI3, MAI1), Asian (PXO99A, KACC 10331) or American (X11-5A) *Xoo* strains as well as African (e.g. MAI10) or Asian *Xoc* strains (e.g. BLS256). As shown in Fig. 2A and Supplementary Fig. S6, *X. oryzae* strains were broadly capable of eliciting xisRNA accumulation with contrasting strength and specificities. In qualitative terms, African *Xoo* and *Xoc* strains induced all tested xisRNAs with the exception of xisRNA007 which seems to be specific to African *Xoo*. The Asian *Xoo* strains and the American *Xoo* strain were less active and fainter but reproducible signals could be detected essentially for xisRNA001 and xisRNA002. In the case of PXO99A, these signals were also T3SS-dependent. While xisRNA023 was not tested for the US *Xo* strain X11-5A, xisRNA023 signals appeared to be specific to the *Xoo* pathovar as these sRNAs were readily detected in response to both Asian and African *Xoo* strains (Supplementary Fig. S6).

As a complementary way to establish that xisRNA induction is not limited to African *Xoo* infection, we exploited the dataset generated by Zhao et al*.*^22^ where sRNAs expression temporal dynamics in *Xoo* PXO99 versus mock-inoculated rice leaves was profiled at early time points following leaf clipping inoculation. A second dataset consisting of sRNA sequencing data 24 hpi with the Azacytidine-resistant derivative of PXO99 strain, PXO99A from the Plant MPSS databases^25^ was also included. The DE statistics of experimental sRNA loci whose expression changed in relevant treatment comparisons and that overlapped with BAI3 xisRNA loci are summarized in Fig. 2B. Coverage profiles of sRNA reads in xisRNA loci further indicate that PXO99-induced reads map exactly to the same location as BAI3-induced reads (see coverage plots in our dataverse). In addition to confirming that PXO99 also induces xisRNA accumulation, the higher sensitivity of sRNA-seq applied to leaf clipping assays samples indicates that the most abundant xisRNAs accumulates as early as six hours following inoculation in an assay that more closely recapitulates a natural infection of xylem vessels as opposed to massive infiltration of the leaf mesophyll.

The broad xisRNA induction capacity in the genetic diversity of both *Xo* pathovars emphasizes the relevance of these sRNAs as evolutionary conserved molecular markers of early rice infection events and implies a potential functional role in this process.

### A broader xisRNAs inventory

**Figure 3:**
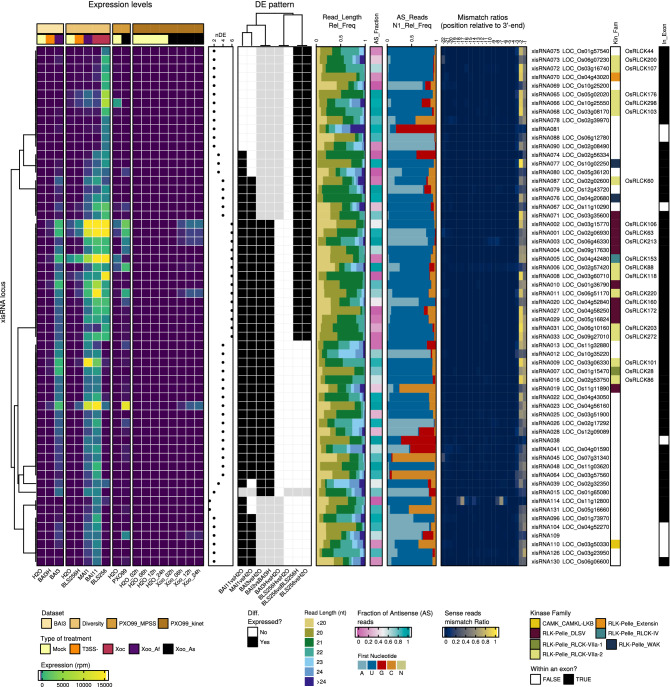
A summary of xisRNAs features in the analyzed sRNA-seq datasets. In the complex heatmap layout, the top 60 xisRNA loci with the highest maximum treatment mean expression across datasets are described row-wise. The first component, on the left, reports on expression (reads per million) in the various inoculation treatments of the different datasets. In the type of treatment legend, the “Xoo_Af” and “Xoo_As” labels stand for African *Xoo* and Asian *Xoo* strains, respectively. The DE patterns component shows whether xisRNA loci were detected as DE in various comparisons. The “nDE” dot plot depicts the number of comparisons where the xisRNA locus was deferentially expressed. Cells in gray indicate that the xisRNA locus was not considered in the dataset because the loci was not detected by ShortStack or because reads in this dataset did not pass our xisRNA loci features criteria. The subsequent plots on the right depicts the size distribution of reads (“Read_Length_Rel_Freq”), the fraction of antisense reads (“AS_Fraction”), the proportion of antisense reads with distinct 5′-end first nucleotide (“AS_Reads_N1_Rel_Freq”) and the specific nucleotide mismatch ratios for relative positions − 22 to − 1 of antisense reads using xisRNA loci mapping reads from the “BAI3” and “Diversity” datasets. The MSU7 identifier of the gene overlapping with a xisRNA duplex locus is indicated together with the kinase subfamily into which the encoded protein is classified (“Kin_Fam.” column) as well as the RLCK protein name, when relevant and available. Finally, the “In_Exon” column indicates whether the xisRNA duplex loci is fully included in an exon of an annotated mRNA of the gene. All numerical values reported in the graphic were computed using reads mapping to the xisRNA duplex loci. Note that the antisense polarity is defined relative to the orientation of the underlying *cis*-gene. To compute values that incorporate a notion of polarity for xisRNA loci that do not overlap with an annotated gene, reads mapping to the top strand were arbitrarily considered to be in the sense orientation.

Having established that xisRNA accumulation is a widespread molecular feature of rice infection by *Xo* pathogens raised the question of whether other strains induced novel xisRNA loci that were not captured by our initial BAI3-specific sRNA-seq experiment. To address this, we performed a second set of rice sRNA-seq experiments, designated as the ‘Diversity’ dataset. African *Xoo* MAI1 strain was selected because in northern blot experiments it strongly induced xisRNAs. As representatives of the *Xoc* pathovar, the African BAI11 strain and the Asian BLS256, together with a T3SS mutant derivative (BLS256H), were also selected to investigate T3SS-dependency and *Xoc* pathovar specific xisRNAs. The setup of these experiments as well as the outcome of the primary genome segmentation and DE analysis are summarized in Supplementary Fig. S7 and Supplementary Table S2. Across both datasets, a total of 163 distinct loci met the criteria defined for xisRNA loci, 99 of which were not identified in the first ‘BAI3’ dataset. Supplementary Table S3 records expression measures, features of mapping sRNA reads and annotated *cis*-genes information for these loci while Fig. 3 depicts a graphical representation of this data for the 60 most highly expressed xisRNA loci. The clustering of DE patterns across comparisons (Fig. 3 and Supplementary Fig. S8) is complex but pointed to few main classes of xisRNA loci: 16 are broadly induced by all tested *Xo* strains (e.g. xisRNA01, 02), 22 are induced by African *Xoo* and *Xoc* strains (e.g. xisRNA009, 22) and 57 are specifically induced by the Asian Xoc BLS256.

In order to ascertain again that the sRNA reads mapping at dataset-specific xisRNA loci in this extended inventory were congruent with the hypothesis that xisRNAs derive from a duplex of sRNAs molecules, we inspected the coverage of all reads from both datasets. With the exception of xisRNA094 for which the shape of read coverage is not consistent with a sRNA duplex model, all other loci displayed a single major ~ 25 bp wide coverage peak. We therefore defined these shorter genomic intervals as ‘xisRNA duplex loci’ (see xisRNA loci-specific genome browser views in our dataverse). As shown in Supplementary Fig. S8 and Fig. 3, duplex loci reads complementary to the *cis*-gene strand have a proportion of 5′-T that is well above the proportion of Ts in the rice genome. A hallmark of sRNA loaded into rice AGO1s complexes is a strong sequence bias for 5′ Uracil^26^. This implies that the composition of *cis*-gene antisense xisRNA 5′-nucleotide is in line with their possible loading into an AGO1 complex.

The joint analysis of our sRNA-seq datasets generated a larger collection of xisRNA loci detected independently with diverse *Xo* strains and confirmed that the main features of xisRNAs described in the BAI3 dataset were also conserved in the Diversity dataset. Furthermore, *cis*-gene antisense xisRNAs might be sorted into OsAGO1 complexes to silence *cis-*genes.

### Many protein products of loci overlapping with xisRNAs harbor kinase domains and are involved in immune responses signaling

**Figure 4:**
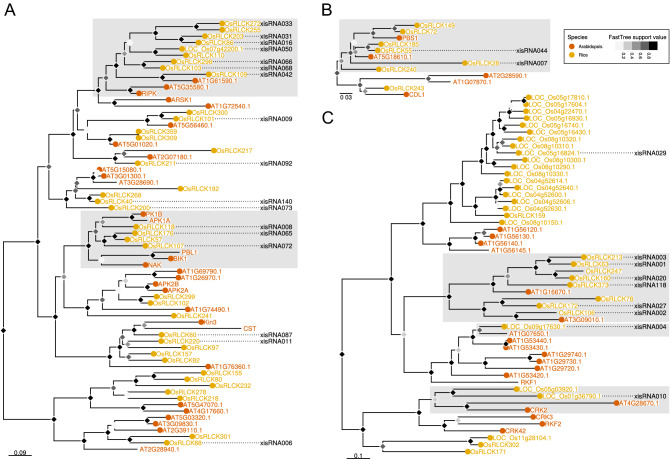
Multiple xisRNA loci *cis*-gene products with kinase domains are related to immune signaling pathways components in rice or Arabidopsis. The displayed phylogenetic trees correspond to selected sub-clades of proteins belonging to the RLCK-VIIa-2 (**A**), RLCK-VIIa-1 (**B**) and DLSV (**C**) subfamilies in the genome-wide phylogenetic tree (Supplementary File S1) constructed using the kinase domain of annotated proteins in the *Arabidopsis* Col-0 and Rice Nipponbare genomes. Dotted lines connect plant protein names with xisRNA identifiers when the xisRNA duplex loci is fully included in an exon of the underlying gene. Shaded rectangles highlight clades that are discussed in the text. The FastTree support value grey scale for tree nodes corresponds to local bootstrappings used by FastTree to estimate the tree's reliability. Scale bars correspond to substitutions per site.

Of the 163 xisRNA duplex loci, 91% (149/163) overlapped with an MSU annotated gene (*cis*-gene) and 75% (122/163) were included within an annotated exon. To examine if those genes had related functions, we performed Plant GO Slim term enrichment analysis (Supplementary Fig. S9). In the Molecular Function ontology domain, we obtained a significant enrichment (FDR ≤ 0.05) of the “RNA binding” term. Among several predicted proteins with RNA-binding domains, the presence of two genes involved in sRNA silencing pathways was especially noteworthy: the ARGONAUTE family member *OsAGO13*^16^ associated with xisRNA064 and the DICER-LIKE family member *OsDCL4*/*SHO1*^27, 28^ associated with xisRNA022 (Fig. 3, Supplementary Table S3). In the Molecular Function GO category, the most significantly enriched terms are connected to “receptor activity” and “kinase activity”, with 64 xisRNA loci overlapping genes (46%) annotated with this latter term. Consistent with GO enrichment analysis, the proportions of the different kinase groups in the xisRNA set is different from the whole genome composition (two-sided Fisher’s Exact test p-value = 5e−04) and a one-sided post-hoc Fisher’s exact test indicated that 5 subgroups (Extensin, WAK, DLSV, RLCK-IV, RLCK-VIIa-2) of the RLK/Pelle protein kinase family are significantly enriched in the xisRNA *cis*-gene products list (Holm method adjusted p-values < 0.05). Subfamilies such as DLSV, WAK, and RLCK-VIIa, were previously shown to be enriched in genes with a role in biotic stress responses based on either their function or expression profile^9, 29^. To investigate in greater details if xisRNA *cis*-genes coding for kinase domain proteins are likely to participate in rice responses to biotic interactions, we constructed a phylogenetic tree of *Arabidopsis thaliana* and rice kinase domain-containing proteins (Supplementary Data S1). In this tree, we identified those instances where a xisRNA duplex locus was fully included within the mature rice transcript. As illustrated in Fig. 4, manual examination of individual subclades with associated xisRNAs revealed some interesting insight: first, 17 xisRNA have a *cis*-gene product in a subtree of RLCK-VIIa proteins (Fig. 4A). Most of the OsRLCK clustering with RIPK, an *Arabidopsis* RLCK involved in ETI signaling, stomatal defense and root development^30^, have a cognate xisRNA. Still on Fig. 4A, in the BIK1/PBL1^30^ clade, OsRLCK107, OsRLCK118 and OsRLCK176 have been previously shown to contribute to PTI or resistance to *Xoo* downstream of the OsCERK1, SDS2 or Xa21 receptors^31–33^ and their transcript has a xisRNA duplex. Second, on Fig. 4B, the RLCK-VIIa proteins OsRLCK28 and OsRLCK55, cluster with the Arabidopsis decoy PBS1^34^ and their mRNA overlap respectively with xisRNA007 and xisRNA044. OsRLCK55 and OsRLCK185 (also in this clade) interact with a *Xoo* type III effector. OsRLCK185 is essential for PAMP-triggered rice immune signaling^35^. Finally, a couple of the most highly and broadly induced xisRNAs are associated with subgroups of RLK/Pelle protein kinase of the DLSV subfamily (Fig. 4C): the xisRNA010-associated protein kinase domain is closely related to the *Arabidopsis* CRK2 protein which is required for PTI and defense against a bacterial pathogen^36^. xisRNA004-associated protein kinase domain is closely related to *Arabidopsis* AT1G07650/LMK1 which possesses cell death induction activity^37^.

Overall, xisRNA duplex loci *cis*-genes are remarkably biased for loci encoding RLK and RLCK proteins. A number of protein sequences harboring a kinase domain and associated with xisRNA loci are phylogenetically or functionally linked to immune signaling. Thus, if xisRNA have regulatory activity, it is conceivable that they act in *cis* to dampen PTI signaling.

### xisRNAs accumulation is dependent on canonical components of the rice regulatory sRNAs biogenesis machinery

As a first step in characterizing the molecular mechanisms underpinning xisRNA induction, we investigated the capacity of rice lines compromised for the activity of canonical regulatory sRNA synthesis pathway components to produce xisRNAs in response to bacterial infection.

**Figure 5:**
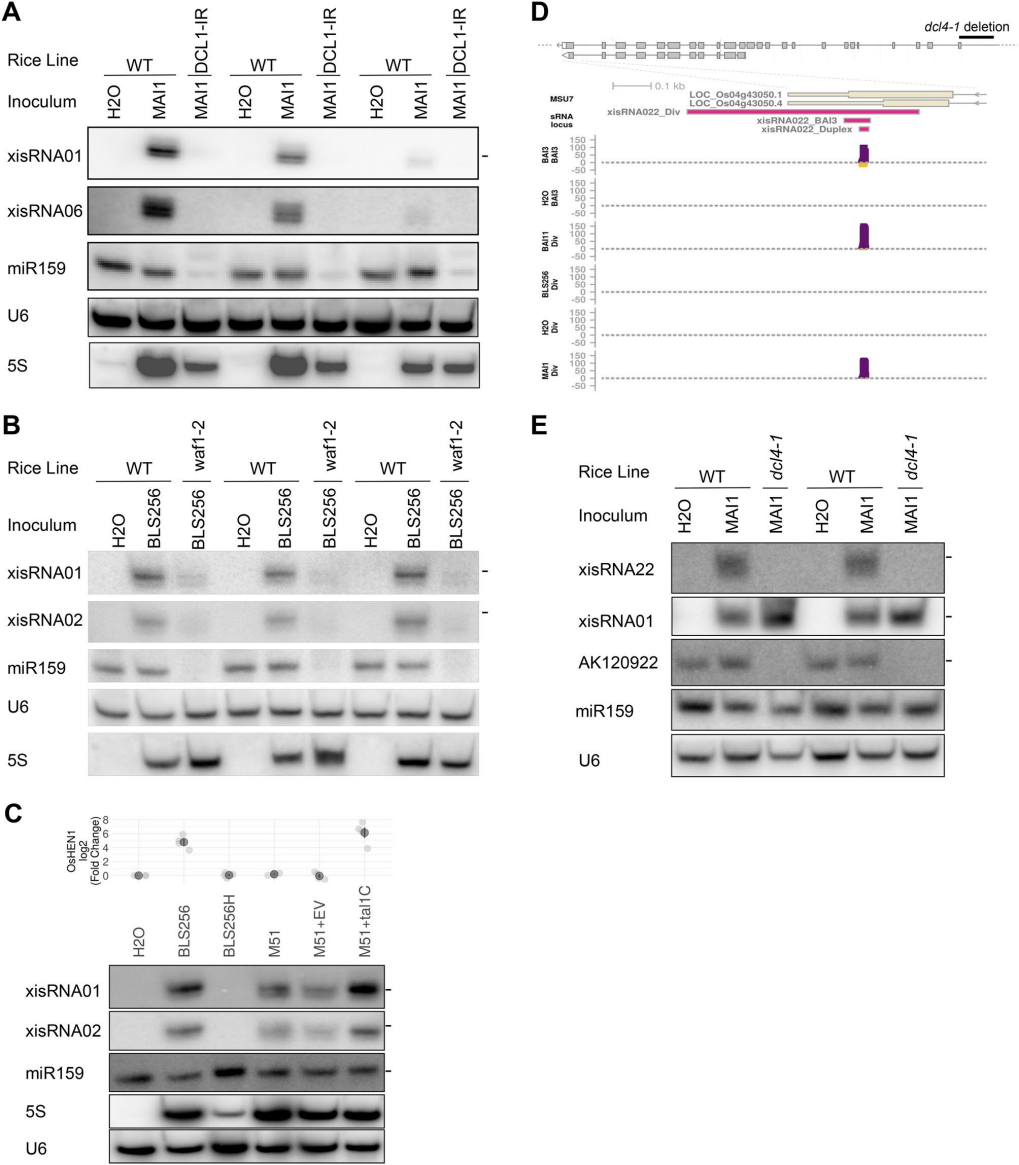
Rice sRNA biogenesis genes impact xisRNA accumulation. The images of autoradiographs obtained after RNA gel northern blot analysis conducted with specific oligonucleotide probes hybridized to total RNA extracted from leaves of wild type Nipponbare (WT) or a *OsDCL1*-silenced line (DCL1-IR) in (**A**), wild type Kinmaze (WT) or a *OsHEN1* mutant allele line (*waf1-2*) in (**B**) or Nipponbare in (**C**). Leaves were infiltrated with the indicated strains and collected 24 hpi. These results are representative of two (*OsDCL1*) and three (*OsHEN1* and Tal1c blots) replicate experiments. In (**C**), the plot on top of the gel images represent *OsHEN1* Q-RT-PCR expression data from four experiments (light gray point) with mean (dark gray points) and standard error (line ranges). The BLS256H strains correspond to a T3SS minus mutant derivative of BLS256. The “+” sign indicate that the strains were transformed with a plasmid expressing Tal1c or the corresponding empty vector (EV). Full-length blots are presented in Supplementary Fig. S15. (**D**) Diagram depicting the general exon–intron structure of *OsDCL4* and a close-up genome browser view of the last exon of the gene with the 3′ UTR. The *dcl4-1* deletion is not drawn to scale but span ~ 1.5 kb on the 5′-side of the gene which includes upstream promoter sequences up to the first 72 bp of exon 1^27^. The genome view represents reads coverage in various treatments, the MSU7 annotation, and the xisRNA022 locus defined in the “BAI3” and “Diversity” sRNA-seq datasets as well as its duplex locus. For details see the legend of Fig. 1F. (**E**) Northern blot analysis conducted on total RNA extracted at 48 hpi from leaves of homozygous wild type (WT) or homozygous *dcl4-1* individuals. This experiment was repeated three times with similar results. In (**A**), (**B**) and (**E**), lanes with the same labels correspond to biological replicates of total RNA samples extracted from a single infiltrated leaf area from individual independent plants. The hyphens on the right of individual blots mark a size of 21 nt. For each panel, individual blot images derive from successive stripping, hybridization and detection rounds of the same membrane.

In rice, OsDCL1 is required for rice canonical ~ 21 nt miRNA biogenesis^38^. Because xisRNAs are 20–22 nt long, we tested the inverted repeat transgenic line OsDCL1-IR previously shown to be specifically silenced for the *OsDCL1*^39^. In northern blots, as expected in plants with *OsDCL1* activity defects, the MAI1-inoculated OsDCL1-IR individual plants had strongly reduced miRNA159 levels (Fig. 5A). The OsDCL1-IR plants also failed to respond to MAI1-inoculations and displayed xisRNA01 and xisRNA06 levels similar to water inoculated negative controls, which suggests that *OsDCL1* is required for the induction of these xisRNAs. We note that in the OsDCL1-IR individuals, the signals for the *X. oryzae* 5S rRNAs probe, acting as a proxy for MAI1 cells leaf density, tended to be lower than in Nipponbare controls. This indicates that *OsDCL1*-silenced plants may be more resistant to MAI1 infection.

Canonical plant regulatory sRNA biogenesis pathways converge on the HEN1 methyltransferase^3^. The rice *wavy leaf1-2* (*waf1-2*) line with a null mutation in *OsHEN1* has been shown to be impaired in the accumulation of miRNAs and ta-siRNA^40^. To investigate whether *OsHEN1* activity is important for xisRNAs biogenesis, we infiltrated wild type Kinmaze and *waf1-2* individual plants with BLS256 and monitored xisRNA accumulation (Fig. 5B). The miR159 probe confirmed that the *waf1-2* mutation greatly reduced miR159 accumulation in homozygous mutant individuals. While BLS256 infiltration triggered xisRNA001 and xisRNA002 accumulation in wild type plants relative to water controls, this effect was very much attenuated in the *waf1-2* individuals indicating that, in addition to *OsDCL1*, *OsHEN1* activity is necessary for xisRNA induction. *OsHEN1* is probably universally upregulated by Asian *X. oryzae* TALEs including Tal1c from the Xoc BLS256 strain and Tal9a from the Xoo PXO99 strain^41,42^. To date, the functional significance of this induction remains elusive. We therefore asked whether Tal1c-mediated *OsHEN1* induction during BLS256 infection affects xisRNA accumulation. As exemplified in Fig. 5C, compared to the wild type BLS256 strain, the xisRNA001 and xisRNA002 signals were slightly weaker in rice leaves infiltrated with the *tal1c* insertion mutant strain M51^41^ in the BLS256 background. Conversely, expression of *tal1c* from a plasmid in the M51 background not only restored *OsHEN1* induction but also increased xisRNA001 and xisRNA002 levels relative to M51 complemented with an empty vector. Because the effect of *tal1c* activity on xisRNA accumulation were relatively mild and to test if *tal9a* has a similar effect, we produced a PXO99A strain derivative with a deletion of the entire tal9 TALE genes cluster, which contains 5 *tal* including the *tal9a* gene^43^. In an experiment summarized in Supplementary Fig. S10, while this mutant had lost the ability to induce *OsHEN1* and this ability could be restored by the *Tal1c* plasmid, xisRNA023 levels were not diminished in leaves infiltrated with the tal9 cluster deleted strains relative to PXO99A. Thus, we conclude that *OsHEN1* induction during *Xoc* BSL256 infection maximizes xisRNA accumulation but a comparable effect is not observed during *Xoo* PXO99A infection.

Overall, our epistasis analysis agrees with the view that xisRNAs production requires the canonical regulatory sRNA biogenesis pathways component *OsDCL1* and *OsHEN1*. As bona fide products of these pathways, xisRNAs may thus possess PTGS-related regulatory activity.

### xisRNA accumulation is DCL4-independent but probably requires expression of the *cis*-gene

OsDCL4 is the preponderant rice DICER-LIKE responsible for the cleavage of dsRNA precursors that releases ~ 21 nt siRNAs associated with inverted repeat transgenes, hp-siRNAs and *TAS3* ta-siRNAs^27^. As noted above and shown in Fig. 5D, the African *X. oryzae*-specific xisRNA022 duplex is located on the last exon of LOC_Os04g43050 which precisely corresponds to *OsDCL4*/SHO1^16^. To test if *OsDCL4* is also involved in xisRNA synthesis, we examined the effect of the null *dcl4-1* allele on xisRNA accumulation in northern blot experiments. The *dcl4-1* mutation, identified in the L16S indica variety, carries a ~ 1.5 kb deletion (Fig. 5D)^27^. The AK120922 transcript can form a hairpin-like structure and has been shown to be processed by OsDCL4 to produce 21 nt hp‐siRNAs^27^. A northern blot oligonucleotide was designed to detect one of the most abundant reads on the genomic region of AK120922 in our BAI3 dataset and was used as a probe to monitor AK120922-derived hp‐siRNAs. As shown in Fig. 5E, while wild type and *dcl4-1* leaves expressed similar levels of miRNA159. The AK120922 signal detected in wild type individuals was lost in samples from the *dcl4-1* plants, thus confirming the absence of OsDCL4 activity. The *dcl4-1* mutation did not impair xisRNA01 accumulation because the corresponding signal in MAI1 infiltrated mutant plants was not decreased relative to corresponding wild type plants. In contrast, MAI1-triggered accumulation of xisRNA022 in *OsDCL4* plants, disappeared in *dcl4-1* individuals.

From these results, we conclude that *OsDCL4* does not play a general role in xisRNAs production. However, the promoter region deleted in *dcl4-1* is specifically required for induction of the *OsDCL4*-associated xisRNA locus. *dcl4-1* plants fail to express a *OsDCL4* transcript^27^ which potentially suggests that the expression of the protein-coding mRNA overlapping with the xisRNA duplex is required for double-stranded precursor biogenesis and consequently the production of the corresponding xisRNA.

### Genome-wide transcriptomics data provides limited support for a general role of xisRNAs in *cis*-loci silencing

We have established that xisRNA loci rely on components of regulatory sRNA biosynthesis pathways to form duplexes of 20–22 nt molecules that predominantly map to exons of protein coding genes. It is therefore conceivable that xisRNAs act in *cis* to silence overlapping transcripts. To address the potential *cis*-regulatory activity of xisRNAs, we applied a standard transcriptomics data analysis approach based on the postulate that candidate target genes are expected to be downregulated in a treatment comparison where the corresponding sRNA is upregulated. We generated paired-end mRNA-seq data using samples from the set of experiments that were also used to obtain the BAI3 sRNA-seq dataset. In addition, we included sequences from Nipponbare samples collected 24 h after infiltration with the *Xoo* MAI1 strain and a water control from a previously published dataset^44^. Finally, sequences from Nipponbare samples collected 48 h after infiltration with the *Xoc* strains BLS256, BAI11 (a.k.a. CFBP7342), or the mock control were also retrieved from another published^45^ dataset. Differential expression metrics were computed for comparisons involving samples inoculated with a virulent strain versus samples inoculated with the corresponding T3SS mutant strain or mock treatment.

**Figure 6:**
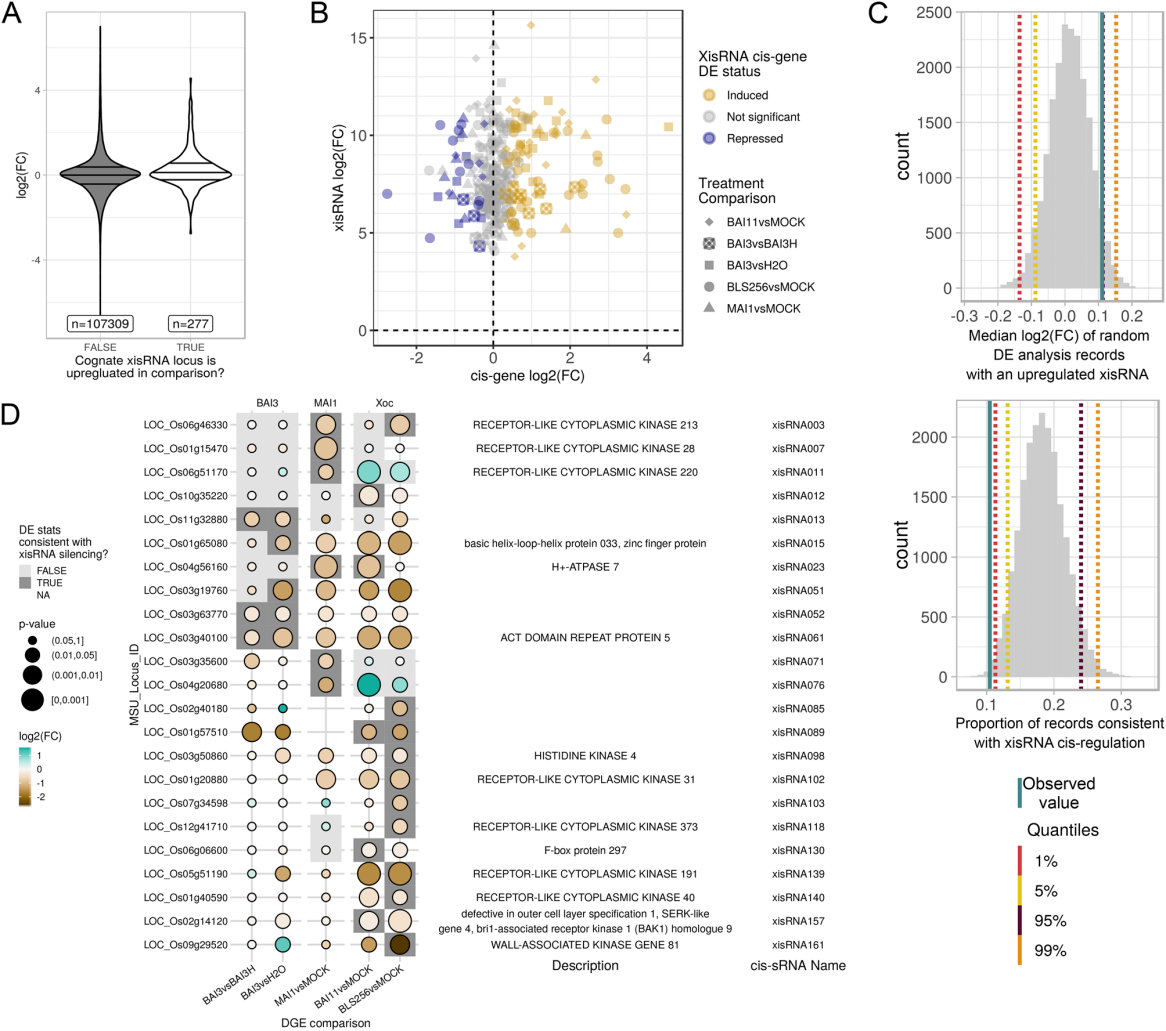
mRNA-seq data analysis points to a restricted set of xisRNA *cis*-genes as potential silencing targets. (**A**) Distribution of the log_2_ transform of the fold change ratio for DE analysis records (expressed gene by treatment comparison combinations) contrasted on whether or not the gene has a cognate xisRNA and this xisRNA is also up-regulated in the same treatment comparison. The horizontal lines reflect the 25%, median and 75% quartiles. (**B**) log2(FC) of xiRNA versus cognate *cis*-gene in a relevant treatment comparison. For each treatment comparison, a *cis*-gene is considered to be ‘Induced’ if it has a log2FC > 0 and a p-value ≤ 0.05, ‘Repressed’ if the log2FC < 0 and the p-value ≤ 0.05 and ‘Not significant’ otherwise. (**C**) Histogram distributions of the variables computed in 20,000 trials for randomization test. (**D**) Summary of the rice annotated *cis*-genes whose expression profile is consistent in at least one comparison with a possible negative regulatory effect of a cognate xisRNA. The ‘DE stats consistent with xisRNA silencing status’ is regarded as “TRUE” if in the corresponding comparison, the xisRNA has been detected as induced and if the gene DE record has log2FC < 0 and a p-value ≤ 0.05, “FALSE” if the xisRNA has been detected as induced but the gene DE record does not pass the log2FC or p-value criteria and “NA” (Not Available; white background) otherwise.

First, we examined the distribution of the log2 transform of the fold change ratio of genes in the various comparisons depending on whether an annotated transcript of the gene overlapped with a xisRNA duplex loci that is upregulated in the corresponding comparison (Fig. 6A). If upregulation of a cognate xisRNA generally suppressed *cis*-gene expression, we would expect the median log2(FC) of this set of DE records to be inferior to zero. The computed median log2(FC) is 0.111 and, on the contrary, is significantly superior to zero (one-sided Wilcoxon rank sum test: W = 25,008, p = 8e−06). Likewise, a two-sided Fisher’s Exact test for a specific enrichment of genes with a cognate up-regulated xisRNA in repressed genes (log2(FC) < 0 and p-value ≤ 0.05) across treatment comparisons produced a moderately significant p-value of 0.03 for the BAI11vsMOCK comparison only. Conversely, of the 277 DE records pertaining to genes having an up-regulated cognate xisRNA in the corresponding comparison only 29 (10.5%) meet these criteria for down-regulation (Fig. 6B).

To explore further whether a xisRNA *cis*-gene gene is more likely to be down-regulated when the corresponding xisRNA is up-regulated, we performed randomization tests (20,000 trials) where each xisRNA loci was assigned a random locus identifier as cognate *cis*-gene. The median log2(FC) of DE records of randomly assigned genes with an upregulated xisRNA as well as the proportion of these records that are consistent with negative regulation (log2(FC) < 0 and p-value ≤ 0.05) were subsequently computed as above for the observed values. As shown in Fig. 6C, 95.1% of the median log2(FC) values in the random set were lower than the observed value. Similarly, 99.4% of the proportions of records that are consistent with negative regulation in the random set were higher than the observed value.

Contrary to our initial hypothesis, this analysis argues against the view that xisRNA generally direct *cis*-silencing. It cannot be ruled out however that xisRNA do occasionally act in *cis* to diminish transcript accumulation and we listed in Fig. 6D, 23 *cis*-gene–xisRNA pairs whose DE data is consistent with a regulatory effect.

### xisRNAs duplexes exhibit limited nucleotide sequence similarity but those associated with kinase loci coincide with mRNA regions encoding a kinase active site motif

**Figure 7:**
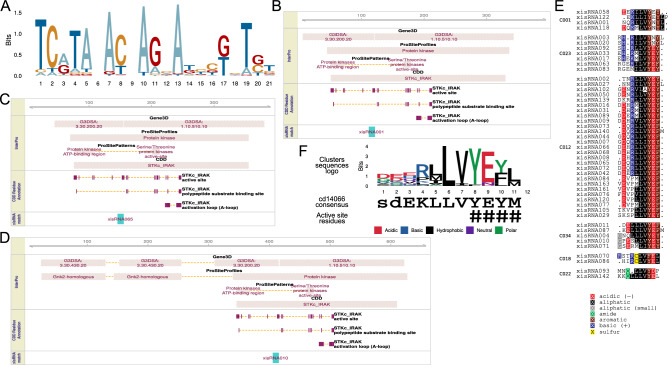
Analysis of xisRNA duplex sequences similarities. (**A**) Sequence logo representation of a multiple alignment of major antisense xisRNA duplex reads sequences belonging to the clusters defined in Supplementary Fig. S11. The sketches of InterPro domain hits in panel (**B)**, (**C**) and (**D**) for respectively xisRNA001, xisRNA065 and xisRNA010 *cis*-gene products are representative and illustrative of the observation that xisRNA duplex coding sequences consistently coincide with a specific kinase domain protein regions. (**E**) Multiple protein subregion sequences alignments for a subset of 6 individual clusters from those defined in Supplementary Fig. S12 (the clusters labels are consistent). (**F**) Sequence logo representation of a multiple alignment of the 47 amino acid sequences displayed in (**E**). The active site residues described for the NCBI CDD STKc_IRAK family (accession cd14066) are highlighted with hash symbols.

The fact that a noticeable fraction of xisRNAs is associated with genes encoding proteins with closely related kinase domains raises the question of xisRNA sequences relatedness. We used systematic pairwise alignments between xisRNA duplex loci sequences of the most abundant read (19–24 nt) mapping on the *cis-*locus antisense strand to perform hierarchical clustering. Of the 148 loci considered (xisRNA133 had no antisense major reads), 54 were grouped into 21 clusters of related xisRNA major reads where the number of variable positions ranges from 1 to 8 (Supplementary Fig. S11). Interestingly, most individual xisRNA duplex loci in these clusters overlap a kinase encoding locus and the sequences clusters display weak overall similarities (relatively lower mismatch counts outside the diagonal of the matrix in Supplementary Fig. S11). To visualize these similarities, the multiple alignment of all major reads in the clusters was used to generate the consensus logo reproduced in Fig. 7A. In line with our earlier observation regarding 5′ nucleotides distribution of antisense reads, major reads frequently start with a “TC” (or “UC” for an RNA sequence) dinucleotide on their 5′-end. The consensus pattern further points to conserved positions that seem to be phased with a 3 nt offset, reminiscent of codon code degeneracy.

This pattern of conserved nucleotides in xisRNA sequences hints to the possibility that xisRNA duplex loci correspond to *cis*-transcripts regions encoding related protein motifs. To determine if xisRNA duplexes correspond to the coding sequences of documented domains, we searched InterProScan hits on *cis*-transcripts translation products and analyzed their overlap with xisRNA-associated protein subsequences (97 out of 122 that overlap an annotated transcript are also strictly within a CDS). Domains that repeatedly overlapped with a xisRNA-associated protein subsequences were connected with kinase activity. For example, 50 out of 97 overlap with a ProSite "Protein kinase" domain (PS50011). As illustrated in Fig. 7B,D, 39/97 xisRNA-associated protein regions overlapped with at least one residue matching with an “active site” features in the NCBI CDD database “STKc_IRAK” domain (cd14066). The corresponding STKc_IRAK conserved sites consensus motif ‘YEYM’ is involved in ATP binding according to its the CDD domain description. As a broader complementary approach, we measured the degree of similarity between translation products of xisRNA duplex loci sequences following a procedure similar to the one used above for xisRNA major reads sequences (Supplementary Fig. S12). While many clusters displayed no particular similarity with one another, a subset of six clusters with a total of 47 sequences appeared to be related as judged by dissimilarity score patterns off the matrix diagonal. In agreement with the InterPro domains overlap analysis, the consensus motif at the C-terminus of the xisRNA-associated protein sub-sequences clusters multiple alignment (LVYE[YF][LM]) match very well with the ‘YEYM’ consensus sequence of the “STKc_IRAK” ATP-binding motif in this region (Fig. 7E,F).

In summary, our analysis of xisRNA duplex antisense sequences similarities revealed that most xisRNAs display limited sequence conservation but defined, nonetheless, a few families containing similar reads. It also appears that about half of the xisRNA duplex overlapping a CDS coincide with sequences coding for a conserved RLK/Pelle kinase motif that is involved in ATP binding.

## Discussion

Our understanding of the biology of plant regulatory sRNAs bears on a few paradigmatic pathways reflecting common biosynthesis routes and regulatory activities. However, computational approaches similar to the one that have been taken here are revealing the hidden diversity of atypical non coding small RNA types^46–48^. Here we report on novel rice 20–22 nt small non-coding RNA populations highly induced upon infection by a set of diverse *X. oryzae* strains. Only pathogenic bacteria possessing the T3SS virulence factor translocation system are competent for xisRNA induction.

The discovery of xisRNAs prompted an examination of rice genetic requirement for their biogenesis. xisRNA production appears to utilize host regulatory small RNA synthesis pathways components *OsDCL1* and *OsHEN1* but not *OsDCL4* (Fig. 5). This suggests that xisRNAs derive from precursors processed by these enzymes and argues in favor of the view that xisRNAs are genuine small silencing RNAs. *OsDCL1* is dispensable for siRNA originating from CentO repeats and inverted repeats transgenes but is strictly required for the production of miRNAs^38^. The ShortStack experimental sRNA loci classification algorithm did not score any of the xisRNA loci as likely *MIRNA.* Our analysis of sRNA reads properties at the xisRNA loci further indicates that upon bacterial infection most of these loci generate sRNA reads mapping to both strands of the genome sequence. These observations are incompatible with the possibility that xisRNA loci correspond to canonical *MIRNA* genes. Previous work has identified differentially accumulating rice miRNA during Asian Xoo infection^21, 22^, it was therefore surprising that no xisRNA locus corresponded to a *MIRNA*.

Even if akin to siRNAs, xisRNA loci reads coverage supports the existence of a single xisRNA duplex at each locus (Figs. 1 and 6A and our dataverse). In contrast, endogenous siRNAs loci such as hp‐siRNAs, phasiRNA, transgene-derived siRNAs, or 24 nt heterochromatic siRNAs generally give rise to several distinct siRNA duplexes that may be phased and that cover more or less uniformly the entire precursor sequence^5^. While xisRNAs do not fit well into canonical classes of plant sRNAs (Table 1), they resemble a subset of DCL1-dependent 21 nt *cis*-nat-siRNAs from rice and Arabidopsis deriving from one specific site in the region of antisense transcripts overlap^49^. If extrapolated to all xisRNA loci, our interpretation that xisRNA022 accumulation requires a sense OsDCL4-encoding mRNA (Fig. 5) is consistent with this view. However, neither the reference rice Nipponbare annotation nor our BAI3-infected leaves mRNA-seq data (see coverage plots in our dataverse) argue for the widespread existence of an antisense messenger transcript complementary to the annotated protein coding mRNA. This antisense pol II RNA transcript could still be a very short-lived intermediate. Alternatively, the antisense strand of the xisRNA dsRNA precursor could be the product of RDR activity (Supplementary Fig. S14).

**Table 1:** A comparison of xisRNA properties against main documented classes of endogenous plant non-coding sRNAs.

sRNA class	Precursor	Preferred DCL	DCL processing duplexes	Size (nt)
het-siRNA	dsRNA dependent on RDR2 and Pol IV	DCL3	Unphased duplexes covering the precursor	23–24
miRNA	Single-stranded hairpin RNA	DCL1	Single duplex	21
hp-siRNAs	Single-stranded hairpin RNA	DCL4	Unphased duplexes covering the precursor	21
phasiRNA	dsRNA dependent on RDR6	DCL4	Phased duplexes covering the precursor	21
NAT-siRNA	Complementary pair of Pol II RNAs forming a dsRNA and often dependent on a RDR	Variable depending on locus (DCL1, DCL2 and/or DCL3)	Variable depending on locus	Variable depending on locus (21, 22 or 24)
xisRNA	dsRNA likely deriving from an underlying Pol II mRNA and an unidentified complementary molecule	DCL1	Single duplex	20–22

Interestingly, polarized signatures of untemplated 3′ terminal nucleotides were detected only in xisRNA reads with a sense orientation relative to the orientation of the overlapping annotated gene (Fig. 1 and Supplementary Figs. S3 and S4). The RDR2 strand of P4R2 RNAs and their DCL3-processing heterochromatic siRNA products carry a single 3′ end untemplated random nucleotide as a result of RDR2 nucleotidyl transferase activity^50^. 3′ to 5′ exonucleolytic truncation and/or 3′ tailing occurs pervasively in sRNAs of an *Arabidopsis hen1* mutant but has also been detected in wild type plants^51^. In this context, the tailing of unmethylated 3′ ends is accomplished by the HESO1 and URT1 uridyltransferases but other types of nucleotide additions occur in a *Arabidopsis hen1-2 heso1-2 urt1-3* triple mutant^52^. The pattern of substitution in xisRNA reads untemplated 3′ nucleotides is biased in favor of cytosines (Supplementary Fig. S4) which would require other nucleotidyltransferases^53^.

In spite of an overall predominance of 20–22 nt reads, our sequencing data indicated that xisRNAs sizes vary remarkably (Fig. 1C). DCL1 dices dsRNA precursor in homogeneously sized 21 nt duplexes except for those 22 nt miRNAs that trigger phasiRNA production. If xisRNAs are indeed *OsDCL1*-dependent, this raises the issue of the origin of xisRNA size variability**.** Just like other plant small RNAs, xisRNA require HEN1 activity for their proper accumulation (Fig. 5B). The presence of noticeable proportions of reads < 21 nt for a few xisRNA loci may indicate that they underwent 3′ to 5′ trimming. This observation and the distinctive preeminence of 3′ non-templated nucleotides in sense reads may further indicate that xisRNA undergo 3′ to 5′ exonucleolytic truncation and that 2′-O methylation by the OsHEN1 methyltransferase is a rate limiting step in xisRNA biosynthesis. The observed effect of OsHEN1 induction by Tal1c (Fig. 5C) that maximizes xisRNA accumulation would be consistent with this conjecture.

Our results about xisRNA features and biogenesis argue in favor of the idea that they possess PTGS-related regulatory activity. The observed enrichment of 5′ uracyl in antisense xisRNAs (Fig. 3 and Supplementary Fig. S8) is a hallmark of sRNAs loaded into OsAGO1 complexes and supports this notion. xisRNA duplex loci generally overlap with annotated rice gene bodies (91%) and are included within annotated exons of these genes (75%). However, our mRNA-seq data analysis provided little support for a general role of xisRNAs in the suppression of *cis*-gene expression (Fig. 6). One potential pitfall of our analysis is that for the MAI1 and BLS256 strains no T3SS mutant inoculated samples were included and we thus may have overlooked PTI-regulated *cis*-genes induced by a T3SS minus strain. To demonstrate xisRNAs silencing activity, future work will need to address whether they are detected in AGO1 complexes and if they act in *trans* through partial complementarity with target transcript or if their regulatory activity mostly lies in translational inhibition of target genes similar to plant 22-nt siRNAs^54^.

xisRNA *cis*-genes are enriched for RLK/Pelle kinase family proteins encoding loci (Fig. 3). In the absence of evidence for a *cis*- or *trans*-regulatory activity, it is difficult to interpret the significance of our results regarding the overlap between the xisRNA duplex loci and the coding sequence of a conserved kinases ATP-binding site (Fig. 7). miRNAs frequently target several members of gene families^2^. For example, the miR482/2118 superfamily targets several plant R genes at the level of a conserved P-loop coding region^6^. Probably evolving under different selective constraints, plant parasitic *Cuscuta* mobile sRNAs can be grouped into superfamilies that also target conserved host protein coding sequences^55^.

We initially identified xisRNAs in experiments with the African *Xoo* strain BAI3 (Fig. 1) but subsequently realized that a subset of xisRNA duplex loci are also induced by other strains of this species and that other subsets of xisRNA duplex loci seem to respond differentially depending on the genetic background of the bacterial strains (Figs. 2, 3 and Supplementary Fig. S6). These observations underscore the importance of this phenomenon in a disease context and implies that it is evolutionary conserved across the *Xo* species diversity and across pathovars causing two distinct diseases. The dependence on a functional T3SS for xisRNA accumulation in a compatible interaction is an indication that xisRNA are functionally connected to rice susceptibility. Although the *in planta* growth defect of a T3SS strain rather than the specific activity of T3SS-dependent virulence effectors could also explain this result, PXO99 triggers xisRNA production at a very early stage (6 hpi) of leaf infection (Fig. 2B) in a T3SS-dependant fashion (Fig. 2A). This approximately correspond to the time frame at which the earliest T3SS virulence effectors dependent effects on the host transcriptome are usually detected^56, 57^. Even if some extent of *Xo* multiplication is conceivably necessary for xisRNA appearance, it is not sufficient as exemplified by several xisRNA that only respond to a subset of virulent strains (Fig. 2A and Supplementary Fig. S6). Consequently, we favor the hypothesis that Xo T3SS virulence effectors are directly implicated in xisRNA biogenesis.

Rice mutant lines in sRNA biogenesis pathways components suffer a range of severe reproductive and developmental phenotypes that preclude rigorous testing in classical susceptibility assays for *Xo* diseases. We showed that Tal1c-mediated *OsHEN1* induction contributes mildly to xisRNA accumulation (Fig. 5C). This indicates that an individual Type III effectors acts on xisRNA production and further describes a first function for this TALE. A BLS256 derivative strain with a *tal1c* mutation is nevertheless as virulent as the wild type^58^. This could be due to an inadequacy of the assay to detect subtle phenotypes or this effect on xisRNA accumulation does not result in altered virulence. In line with previous studies on rice susceptibility to *M. oryzae*^39^, our northern blot data on the *OsDCL1* silenced plants (Fig. 5A) does provide circumstantial evidence that this gene promote BLB susceptibility.

Many of the xisRNA duplex *cis*-genes encoding kinase domain proteins have documented or highly likely functions in rice immune signalling (Fig. 4) but, as discussed above, the level of the *cis*-transcript is rarely reduced in the presence of the cognate xisRNA. There are however exceptions, such as the xisRNA007 *cis*-gene (Fig. 6) that belongs to the OsRLCK185 clade (Fig. 4B). Of the non-kinase loci overlapping xisRNAs, xisRNA023 is probably the most conspicuous. Not only because it is one of the most highly expressed in response to all strains except Xoc BLS256 (Fig. 3), but also because its *cis*-gene, *OSA7*, which is downregulated in the presence of xisRNA023 (Fig. 6), codes for a plasma membrane H^+^-ATPase proton pomp^59^ and is the rice orthologue of *Arabidopsis AHA1* and *AHA2* genes that have important functions in a range of physiological processes including immune signaling^60^.

xisRNAs are evocative of eukaryotic pathogens’ mobile sRNAs that suppress plant immunity genes expression and favor compatibility in Cross-Kingdom RNAi^7^. However, it is doubtful that xisRNA or their precursors are of bacterial origin because their sequences poorly match the genomic sequences of the bacterial triggers (Supplementary Fig. S13). Taken altogether, our results can be formalized in a working model (Supplementary Fig. S14) postulating that *Xo* pathovars use T3SS-translocated virulence effectors to co-opt rice sRNA biosynthesis and activity pathways to produce an unusual class of small silencing RNAs that alter plant immune signaling in order to promote bacterial infection. An alternative working model that appears less likely is that rice generates xisRNAs that direct bacterial gene silencing to mitigate bacterial invasiveness similar to what has been reported for a bacterial plant pathogen^61^ and gut bacteria^62^.

This study describes the discovery of xisRNAs. Future efforts will investigate in greater details their molecular activity and biological function with the long term goal to leverage this basic knowledge to improved disease control strategies in the field.

## Methods

### Bacterial strains and plant inoculation

The *Xo* strains used in this study are described in Supplementary Table S4. The isolates were maintained in 15% glycerol at − 80 °C. All *Xoo* strains were cultivated on PSA (10 g/l peptone, 10 g/l sucrose, 1 g/l glutamic acid, 15 g/l Bacto Agar), supplemented with kanamycin (50 μg/ml), rifampicin (100 μg/ml) and/or tetracycline (5 μg/ml) when needed for the propagation of mutant and complemented strains. For plant infiltration, frozen bacterial stocks were spread on Petri dishes with PSA and incubated at 28 °C. On the morning of the experiment, bacterial suspensions were prepared in distilled water and injected with a 1 ml needleless syringe on the abaxial face of the youngest fully expended leaf of 4 weeks old rice plants. Bacterial suspensions were adjusted at OD_600nm_ = 0.5 except for infiltration of *waf1-2* homozygous plants that were found in preliminary experiments to require a three fold more concentrated suspension in order to deliver an equivalent number of bacteria in the leaf mesophyll at t_0_. After infiltration, plants were kept in the same green-house growth conditions until sample collection.

### Plant material and growth conditions

This study involved only laboratory experiments with cultivated rice parental lines and complied with relevant institutional, national, and international guidelines and legislation. Plants were grown under greenhouse conditions with cycles of 12 h of light at 28 °C and 80% relative humidity and 12 h of dark at 25 °C and 70% relative humidity. The following rice genotypes were used: cultivar Nipponbare^24^, the DCL1-IR transgenic line #43-6 in the Nipponbare background^39^, Kinmaze variety and *waf1-2* mutant derivative^40^ and a heterozygous *dcl4-1* line^27^. For experiments on the effect of *dcl4-1*, the wild type controls consisted of individuals in the progeny of selfed *dcl4-1* heterozygous parents that were genotyped as homozygous wild type at the *OsDCL4* loci whereas *dcl4-1* plants were confirmed to be homozygous for the mutant allele.

For PCR genotyping with primers listed in Supplementary Table S5 and the GoTaq G2 DNA Polymerase (Promega) according to manufacturer’s instructions, template rice genomic DNA was extracted from leaf samples using a standard MATAB‐based protocol^63^.

### RNA extraction

Unless stated otherwise, ~ 2 cm fragments were collected from a leaf of three independently infiltrated plants at the specified time point after inoculation and pooled in 2 ml microtubes containing 3 steel beads and immediately frozen in liquid nitrogen. For routine northern blot and Q-RT-PCR experiment, total RNA was extracted using 1 ml of Tri reagent (MRC) following the manufacturer’s instructions and resuspended in nuclease-free water. For Illumina sequencing, the total RNA was treated with the TURBO DNA-free Kit (ThermoFisher) following instructions from the manufacturer and further purified with the mirVana Isolation Kit (ThermoFisher).

### RNA next-generation sequencing

The sRNA-seq data for the BAI3 dataset was generated by the Fasteris company (Geneva, Switzerland). Briefly, sRNA libraries were prepared with total RNA extracted from duplicate samples collected at 24 hpi using the Illumina TruSeq small RNA kit with polyacrylamide gel purified 18–25 nt sRNA fractions and sequenced on a portion of a Illumina HiSeq 2000 lane, 1 × 50 single-reads. The sRNA-seq data for the Diversity dataset was generated by the Novogene company (Hong Kong, China) from total RNA extracted from triplicate samples collected at 24 hpi using a NEBNext Small RNA Library Prep Set for Illumina kit optimized by Novogene on the supplied total RNAs. Ultimately, size selected 18–47 bp inserts were sequenced on a Illumina HiSeq (1 × 50 bp). The ‘BAI3’ mRNA stranded sequencing was performed on a HiSeq 2000 lane (2 × 100 bp) by the Fasteris company (Geneva, Switzerland) with libraries prepared following a dir-mRNA-dUTP protocol that includes poly-adenylated transcripts selection followed by cDNA library construction using Illumina TruSeq Stranded mRNA Library Prep kit. Demultiplexing of sequencing reads was performed by the sequence providers.

### Northern blots and quantitative RT-PCR

Low molecular weight northern blots were performed using previously described standard procedures^64^. Briefly, 10 μg of total RNA in a volume of 40 μl in 1 × gel loading buffer II (Ambion) were loaded on a 17.5% denaturing acrylamide gel and electrophoresed at 80 V, transferred to a Hybond NX membrane (Amersham) and crosslinked with UV. The membrane was then pre-hybridized 30 min with PerfectHyb buffer (Sigma-Aldrich). The radioactively labeled probe was first prepared with γ[^32^P]ATP and DNA oligonucleotides listed in Supplementary Table S5 with a T4 polynucleotide kinase kit (Promega), purified on MicroSpin G-25 (GE Healthcare) column and denatured 2 min at 95 °C. The RNAs on the membrane were hybridized with the probe overnight at 40 °C in PerfectHyb buffer and washed three times with a 2X SSC 0.1% SDS solution for 5 min. Radioactive signals were imaged using a Typhoon phosphorimager (Amersham). Contrast adjustments were applied to blot hybridized with individual probes in a linear fashion and faithfully represent original signal information. The position of the 21 nt marker was determined by superposing images for the xisRNA blots with the corresponding one for miRNA159. Before hybridization with a new probe, northern membranes were stripped with three 10 min washes in near-boiling 0.1% SDS and re-equilibrated in 2 × SSC.

For RT-qPCR, after contaminant DNA removal with the TURBO DNA-free Kit (ThermoFisher), 1 μg RNA was reverse transcribed into cDNA using SuperScriptIII (ThermoFisher) and random hexamers as specified by the manufacturer. The reactions were diluted 5 times in water before quantitative real‐time PCR on a LightCycler LC480 (Roche), using SYBR‐based Mesa Blue qPCR Mastermix (Eurogentec) and the oligonucleotides primers listed in Supplementary Table S5. Transcript levels in a sample were calculated using the ∆∆Ct method from two technical replicate reactions from the same cDNA preparation using the *OsEF‐1*α gene for normalization.

### Deep sequencing data primary analysis

All primary analysis steps were conducted separately for each individual dataset. The first stage of our sRNA sequencing data processing pipeline included a 3′ adaptor trimming step performed with AdapterRemoval (v. 2.2.2—https://github.com/MikkelSchubert/adapterremoval) using these key parameters : “--mm 5 --minadapteroverlap 3 --minlength 18 --maxlength 50”. Illumina reads were mapped onto the nuclear rice genome with bwa aln^65^ version 0.7.17 with default parameters. sRNA loci inference was performed with ShortStack (v. 3.8.5)^23^ using the following key parameters: “--dicermin 20 --dicermax 24 --mincov 2 rpm --pad 75 --foldsize 400 --strand_cutoff 0.8”. Read counts were obtained using the summarizeOverlaps function from the GenomicAlignments Bioconductor package. Experimental sRNA loci that accumulated less than 30 total reads across all libraries in the dataset were excluded. Likewise, sRNA loci that overlapped with rice loci with feature types "tRNA", "rRNA" or "pseudogenic_tRNA" in the gff annotation file for NCBI RefSeq assembly accession GCF_001433935.1 were excluded from differential expression (DE) analysis.

For replicated datasets, standard DE analysis was performed with the DESeq2 R Bioconductor package^66^. For normalization, size factors were estimated using the “iterate” method and the “shorth” function to compute a location for a sample. Dispersions were fitted using the "parametric", by default, or the "local" method, in case estimates did not converge. Two-tailed Wald test were used for DE tests where the alternative hypotheses was that the absolute value of the log2 transform of the fold change ratio is greater than 2. Adjusted p-values were computed with the Benjamini and Hochberg method and considered significant when equal or below 0.05.

For unreplicated datasets, DE analysis used the DESeq package^67^ with default parameters except that a “blind” value for the method parameter and a value "fit-only" for the sharingMode parameter were applied instead in the calls to the estimateDispersions function. As above, dispersions were fitted using the "parametric", by default, or the "local" method, in case estimates did not converge. Benjamini and Hochberg adjusted p-values were computed for the null hypothesis that the value of the log2 transform of the fold change ratio is equal to 0. The latest version of our primary sRNA processing workflow was written into a Snakemake pipeline and is available on gitHub (https://github.com/Aucomte/sRNAmake).

For the analysis of differential gene expression using mRNA-seq data, we applied the ARMOR pipeline^68^ with the following parameter values specified in the config file: additional_salmon_index: "-k 31", additional_salmon_quant: "--seqBias --gcBias". Note that whenever available in the sample metadata information, a ‘experiment’ term was included in the statistical model for sRNA loci and gene DE analysis in order to block for experimental batch effects.

### Secondary bioinformatic analysis

Rice Nipponbare genomic data^24^ including genome sequence, gene annotation and transcripts sequences were downloaded from the MSU website. MIRNA annotation was obtained from MirBase (v22)^69^. Unless otherwise stated, pri-miRNAs loci considered in these analysis correspond to those reference rice sRNA loci annotations in the Plant small RNA gene annotations database^46^ that are flagged as “MIRNA” (“type” field), that are also listed in miRBase (“source” field has value "miRBase21") and for which an overlapping experimental sRNA loci has been identified in all the datasets considered in this study.

We performed Singular Enrichment Analysis for GO Slim Gene ontology terms in the “Rice MSU7.0 nonTE gene” dataset on the AgriGO website (http://bioinfo.cau.edu.cn/agriGO/—date: 10/09/2020) with the list of *cis*-gene identifiers and default parameter values (Fisher statistical test, Yekutieli p-value correction).

xisRNA *cis*-gene kinase annotation in Fig. 3 and Supplementary Table S3 was based on the kinase classification of Lehti-Shiu and Shiu^9^ and the rice OsRLCK gene family classification of Vij et al.^70^.

To build the rice and Arabidopsis kinase phylogenetic tree available in Supplementary Data S1, the hmmsearch program^71^ was used to search for Hidden Markov Model (HMM) kinase profile (PF00560) into the rice MSU7 and Arabidopsis TAIR10 proteomes. Kinase domain sequences were aligned using MAFFT^72^. This alignment was cleaned with TrimAl configured to remove sites with more than 80% of gaps^73^. Based on this alignment, a phylogenetic tree was computed with the Fasttree method (approximately-maximum-likelihood method)^74^.

The search for domain hits in the InterPro database^75^ used InterProScan linux executable and dataset (v. 5.41-78.0) as well as the Panther data (v. 14.1) with default parameters on the set of MSU7 annotated protein with an underlying xisRNA duplex region fully included within the coding sequence.

The search for major xisRNA reads similarities in bacterial genomes used the blastn tool of the BLAST + suite (v. 2.10.1) with these relevant parameters: "-task \"blastn-short\" -max_target_seqs 5 -perc_identity 80". The blast database was created with the following strain and NCBI assembly accessions: BAI3 (GCA_003031385.1), MAI1 (GCA_003031365.1), BLS256 (GCA_000168315.3), BAI11 (GCA_000940825.1).

The secondary bioinformatic and statistical analysis that generated the graphics and results included in this study have been essentially conducted using the R Statistical programming language and packages, notably from the Bioconductor project^76^. The corresponding code and output files have been deposited in a dataverse with the 10.23708/RXIXM3.

## Supplementary Information

Supplementary Information 1.

Supplementary Information 2.

Supplementary Information 3.

## Data Availability

Deep sequencing data was downloaded from SRA using the following NCBI BioProject accessions: ‘MAI1’ mRNA-seq dataset (PRJNA427491), ‘Xoc’ mRNA-seq dataset (PRJNA280380), PXO99 infection time course sRNA-seq dataset (PRJNA252424). The PXO99A sRNA-seq dataset was downloaded from the Plant MPSS databases (https://mpss.danforthcenter.org/web/php/pages/library_info.php?SITE=rice_sRNA&showAll=true). The sequences generated in the course of this study were deposited to SRA under the following NCBI BioProject accessions: ‘BAI3’ sRNA-seq dataset (PRJNA552731), ‘Diversity’ sRNA-seq dataset (PRJNA552775), ‘BAI3’ mRNA-seq dataset (PRJNA679478).

## Code availability

The R code used to generate the material described in this study as well as the R session file and additional plots are stored in a dataverse and persistently available at https://doi.org/10.23708/RXIXM3.
