# Supplementary material for: An atypical class of non-coding small RNAs is produced in rice leaves upon bacterial infection
Source: Sci Rep. 2021 Dec 17;11:24141. doi: 10.1038/s41598-021-03391-9 (PMC8683429; doi:10.1038/s41598-021-03391-9)
Supplement: Supplementary file 3 — Supplementary Information 3. [file 41598_2021_3391_MOESM3_ESM.pdf]

An atypical class of non-coding small RNAs is produced in rice leaves upon bacterial infection

Ganna Reshetnyak‡[1], Jonathan M. Jacobs‡[1,2], Florence Auguy[1], Coline Sciallano[1], Lisa Claude[1], Clemence Medina[1], Alvaro L. Perez-Quintero[1,3], Aurore Comte[1], Emilie Thomas[1], Adam Bogdanove[4], Ralf Koebnik[1], Boris Szurek[1], Anne Dievart[5,6], Christophe Brugidou[1], Severine Lacombe[1], Sebastien Cunnac[1]*

[1] PHIM Plant Health Institute, Univ Montpellier, IRD, CIRAD, INRAE, Institut Agro, Montpellier, France

[2] Present address: Department of Plant Pathology, The Ohio State University, Columbus, OH, United States, USA 43201 and Infectious Disease Institute, The Ohio State University, Columbus, OH, United States, USA 43201

[3] Present address: Department of Agricultural Biology, Colorado State University, Fort Collins, CO, USA

[4] Plant Pathology and Plant-Microbe Biology Section, School of Integrative Plant Science, Cornell University, Ithaca, NY, United States

[5] UMR AGAP Institut, Univ Montpellier, CIRAD, INRAE, Institut Agro, F-34398, Montpellier, France.

[6] CIRAD, UMR AGAP Institut, F-34398, Montpellier, France.

‡ The first two authors should be regarded as Joint First Authors

* Corresponding author

SUPPLEMENTARY FIGURE LEGENDS

Supplementary Figure S1: BAI3 dataset analysis flowchart and main features

Sketch of the overall experimental and bioinformatics procedures used for the production and analysis of the sRNA-seq data for the BAI3 dataset. Bulleted list elements correspond to the main steps in the workflow. The first dotplot depicts the total number of reads included in the DE analysis broken down by replicate sample (grey color) and treatment. The red colored symbols in the MA plots correspond to loci detected as DE in the relevant comparison. Similar to a Venn diagram, the "Upset plot" summarizes the cardinality of the intersection between various combinations of sets of DE sRNA loci in treatment comparisons.

Supplementary Figure S2: Size distribution plots and Genome browser views for control *MIRNA* loci.

The pri-miRNAs loci considered in this analysis correspond to those reference rice sRNA loci annotations in the Plant small RNA gene annotations database (1) that are flagged as "MIRNA" and for which an overlapping experimental sRNA loci has been identified in all the datasets considered in this study. The Dicer Call (DC) flag values (20, 21, 22 or 24) included in the database are taken as indicative of the overall general size of these miRNAs in rice libraries.

A. Distribution of the the fraction of reads by length for pri-miRNAs loci as a function of their Dicer Call in the Plant small RNA database. One data point is the proportion of reads of the corresponding length mapped to an individual pri-miRNAs loci and deriving from libraries constructed with BAI3 inoculated leaves.

B. Similar to A but represents size proportions as stacked bar plots for selected pri-miRNAs loci. The total number of reads from the libraries considered to compute the proportions at individual loci are printed on the bars. Columns in the plots grid group together pri-miRNAs loci by Dicer Call values and the rows correspond to the experimental treatment.

C. Genome browser snapshots of a few selected pri-miRNA loci with their miRBase annotation and read coverage tracks (reads per million) for the various experimental treatments. Coverage of reads mapping to the top genomic strand is represented with positive values and a violet colored area whereas coverage of reads maping to the opposite strand is represented with negative values and a golden colored area.

Supplementary Figure S3: Genome browser snapshots with strand specific read alignments for selected xisRNA loci.

Individual panels span the genomic loci as defined by Shortstack in the 'BAI3' dataset for xisRNA02 (**A**), xisRNA23 (**B**), xisRNA01 (**C**) and xisRNA09 (**D**). Individual tracks correspond to reads from the various bacterial treatments conditioned on the mapping strand on the genome ('+' or '-' prefix on the track title). Read positions with a color different from black indicate a mismatch

with the genomic sequence at these locations. The three downward pointing arrowheads at the bottom of some of the tracks indicate that the plotting area was too short to display all reads.

Supplementary Figure S4: BAI3 xisRNA reads mapping to the same strand as the annotated transcript exhibit specific 3'-end mismatches.

A. Nucleotide substitution matrix of mismatches for positions -1 and -2 relative to the 3'-end of reads from the BAI3 treatment mapping to a xisRNA loci and whose polarity is the same as the annotated *cis*-gene. The counts correspond to the total number of read *vs* genome nucleotides comparisons that yielded the corresponding substitution pattern. Comparisons where nucleotides matched are not included in the matrix.

B. Distribution of genomic sequence mismatch ratios along relative positions within reads for control experimental sRNA loci. This plot is similar to panel I of Figure 1 although in this case the mismatch ratios were computed for a set of 190 control experimental sRNA loci defined in the BAI3 dataset. They were selected based on the following criteria: they are not xisRNA loci, they overlap with a rice sRNA loci of the Plant small RNA gene annotations database (1), they overlap on at least 20bp with an MSU7 annotated exon, their ShortStack Dicer Call is between 20 and 23, and their ShortStack 'FracTop' values is between 0.1 and 0.9. Each data point represents the ratio of the number of mismatches over the total number of comparisons with the genomic sequence for that position of all the reads from the BAI3 treatment and mapping to a sRNA loci of the control set in the sense orientation (violet) or antisense orientation (golden) relative to the MSU annotated gene overlapping with the sRNA loci. None of the two-sided Wilcoxon signed rank tests measuring differences in sRNA mismatch ratios between read orientations performed at each position were significant (BY adjusted p-value ≤ 0.05). The bar graph on the top reports on the total count of comparisons for each orientation.

C. Similar to panel B except that the colored points represent mismatch ratios for the same control *MIRNA* loci as those described for Supplementary Figure 2A (golden) versus xisRNA loci defined in the BAI3 data set (pink). Here, only reads from the BAI3 treatment mapping to the sense strand of the *cis*-gene or the MIRNA loci are considered. Positions where a one-sided Wilcoxon signed rank tests measuring if the mismatch ratios for xisRNA loci is greater than for control MIRNA loci was significant (BY adjusted p-value ≤ 0.05) are marked with a "*" sign. Here, "***" indicate an adj. P-val. ≤ 0.001.

Supplementary Figure S5: Northern blots of a xisRNA accumulation time course experiment.

Autoradiographs obtained after RNA gel northern blot analysis conducted with specific oligonucleotide probes hybridized to total RNA extracted from Nipponbare leaves infiltrated with the indicated strains and collected at the indicated time points (hpi = hours post inoculation). The

BAI3H strains corresponds to a T3SS defective mutant derivative of the BAI3 virulent strain. The xisRNA probes detect RNAs complementary to the sense strand of the xisRNA *cis*-gene. The two columns of images were obtained from two membranes having samples collected from the same infiltration experiment and ran on two separate gels for migration and transfer. Blotting, hybridization and signal detection were performed at the same time and in the same conditions. The hyphens on the right of individual blots mark a size of 21nt. For a given probe, images from both blots are displayed with the same rendering settings (contrast and intensity). Individual images in each column derive from successive stripping, hybridization and detection rounds of the same membrane. Full-length blots are presented in Supplementary Figure S15. This experiment was performed once.

Supplementary Figure S6: xisRNAs are induced by diverse *Xoo* and *Xoc* strains.
Autoradiographs obtained after RNA gel northern blot analysis conducted with specific oligonucleotide probes hybridized to total RNA extracted from Nipponbare leaves infiltrated with the indicated strains and collected 48h post inoculation. The BAI3H strains corresponds to a T3SS minus mutant derivative of the BAI3 virulent strain. The xisRNA probes detect RNAs complementary to the sense strand of the xisRNA *cis*-gene. The two columns of images were obtained from two membranes having samples collected from the same infiltration experiment and ran on two separate gels for migration and transfer. Blotting, hybridization and signal detection were performed at the same time and in the same conditions. The hyphens on the right of individual blots mark a size of 21nt. For a given probe, images from both blots are displayed with the same rendering settings (contrast and intensity). Individual images in each column derive from successive stripping, hybridization and detection rounds of the same membrane. Full-length blots are presented in Supplementary Figure S15. This experiment was performed once.

Supplementary Figure S7: Diversity dataset analysis flowchart and main features.
Sketch of the overall experimental and bioinformatics procedures used for the production and analysis of the sRNA-seq data for the Diversity dataset. Bulleted list elements correspond to the main steps in the workflow. The first dotplot depicts the total number of reads included in the DE analysis broken down by replicate sample (grey color) and treatment. The red colored symbols in the MA plots correspond to loci detected as DE in the relevant comparison. Similar to a Venn diagram, the “Upset plot” summarizes the cardinality of the intersection of various combinations of sets of DE sRNA loci in treatment comparisons.

Supplementary Figure S8: Joint analysis of the BAI3 and Diversity sRNA-seq datasets.
A. Upset plot of the cardinality of the intersection of various combinations of sets of DE xiRNA loci in treatment comparisons across the BAI3 and diversity datasets.

B. Distribution of the proportion of 5' nucleotides at the first position of the reads from the BAI3 and the Diversity datasets computed for individual xisRNA duplex loci depending on whether the reads map to the sense or the antisense strand of the *cis*-gene. Note that for xisRNA loci that do not overlap with an annotated gene, reads mapping to the top strand were arbitrarily considered to be in the sense orientation. The horizontal dashed lines indicate the background proportion of the corresponding nucleotide calculated using the whole rice Nipponbare nuclear genome.

Supplementary Figure S9: Gene Ontology terms enrichment in xisRNA-associated genes.
Only those GO Slim terms with an enrichment test adjusted p-value ≤ 0.05 are displayed.

Supplementary Figure S10: The tal9 region of PXO99A does not contribute to xisRNA accumulation.
OsHEN1 induction Q-RT-PCR data (bar plot) and sRNA accumulation (northern blots) produced with total RNA extracted from Nipponbare leaves infiltrated with the indicated strains and collected 24h after inoculation. The Δtal9 strains correspond to two independent colonies produced by deleting the tal9 cluster in PXO99A. The "+" sign indicate that the strains were transformed with a plasmid expressing *Tal1c* or the empty vector (EV). Full-length blots are presented in Supplementary Figure S15. This experiment was performed once.

Supplementary Figure S11: Sequence similarities among xisRNA major reads.
A. Matrix of distances as computed by the nmismatch function of the Biostrings R package for pairwise global alignments between xisRNA major reads. A 'major read' sequence is the most abundant unique sequence of size between 19 and 24nt from those mapping to a xisRNA duplex loci (at least 15nt included in the duplex) overlapping an annotated MSU7 gene and corresponding to the antisense strand of this *cis*-gene. Rows and columns are ordered based on a Hierarchical cluster analysis. xisRNA sequence clusters membership was determined by cutting the hierarchical tree in order to generate 115 clusters. Clusters that contained at least two xisRNA major reads were labeled (eg C025) on the left and on top of the heatmap. The "Kin. Fam." annotation column reflects the kinase subfamily of the xisRNA locus *cis*-gene.
B. Multiple sequence alignments of the xisRNA major reads within the clusters defined in A.

Supplementary Figure S12: Amino acid sequence similarities of the subregions encoded by xisRNA duplex overlapping coding sequences.
xisRNA duplexes genomic intervals were transposed into positions in the coding sequence space of overlapping annotated transcripts to obtain approximate intervals in protein sequence by mapping CDS positions to codon number. As in Supplementary Figure 11, a matrix of distances and a Hierarchical cluster analysis were performed using the amino acid sequences of the

corresponding protein subregions. Pairwise global alignment distances were computed with the dis function of the bios2mds R package. xisRNA-associated protein subsequences cluster membership was determined by cutting the hierarchical tree in order to generate 44 clusters. Clusters that contained at least two xisRNA-associated subsequences were labeled (eg C001) on the left and on top of the heatmap. Multiple sequence alignments of the amino acid sequence within these clusters is displayed on the left of the heatmap. The “Kin. Fam.” annotation column reflects the kinase subfamily of the xisRNA locus *cis*-gene.

Supplementary Figure S13: The sequence identity of xisRNA major reads with their blast hits in *Xo* genomes sequences.
The blastn executable was used to search hits for all xisRNA major reads sequences (see definition in the legend of Supplementary Figure 11) in the *Xo* genomes listed on the x-axis. The proportion of identical nucleotide (y-axis) was computed by dividing the values of the blast results field "nident” by those of the “qlen” field and only the highest value per query sequence and subject genome was retained to compute the distributions displayed in the box plot.

Supplementary Figure S14: An hypothetical model of xisRNA biogenesis and function. This diagram was drawn using Microsoft PowerPoint 2007.

Supplementary Figure S15: Annotated full-length northern blots images for cropped blots presented in this study

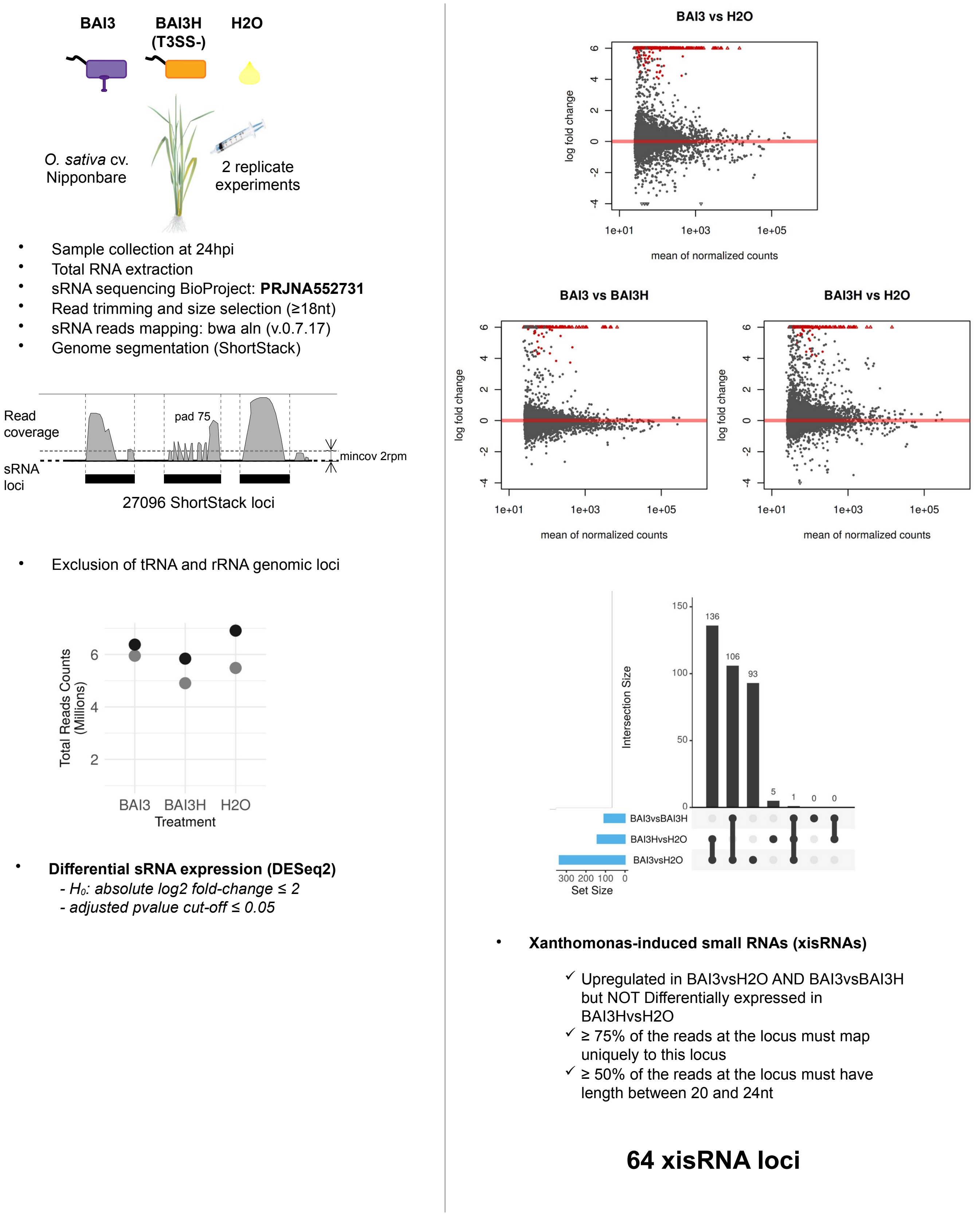
BAI3
BAI3H (T3SS-)
H2O
O. sativa cv. Nipponbare
2 replicate experiments
Sample collection at 24hpi
Total RNA extraction
sRNA sequencing BioProject: PRJNA552731
Read trimming and size selection (≥18nt)
sRNA reads mapping: bwa aln (v.0.7.17)
Genome segmentation (ShortStack)
Read coverage
pad 75
mincov 2rpm
sRNA loci
27096 ShortStack loci
Exclusion of tRNA and rRNA genomic loci
Total Reads Counts (Millions)
BAI3 BAI3H H2O
Treatment
Differential sRNA expression (DESeq2)
- H_0: absolute log2 fold-change ≤ 2
- adjusted pvalue cut-off ≤ 0.05
BAI3 vs H2O
BAI3 vs BAI3H
BAI3H vs H2O
log fold change
mean of normalized counts
Intersection Size
136
106
93
5
1
0
0
BAI3vsBAI3H
BAI3HvsH2O
BAI3vsH2O
Set Size
Xanthomonas-induced small RNAs (xisRNAs)
Upregulated in BAI3vsH2O AND BAI3vsBAI3H but NOT Differentially expressed in BAI3HvsH2O
≥ 75% of the reads at the locus must map uniquely to this locus
≥ 50% of the reads at the locus must have length between 20 and 24nt
64 xisRNA loci

Figure S1

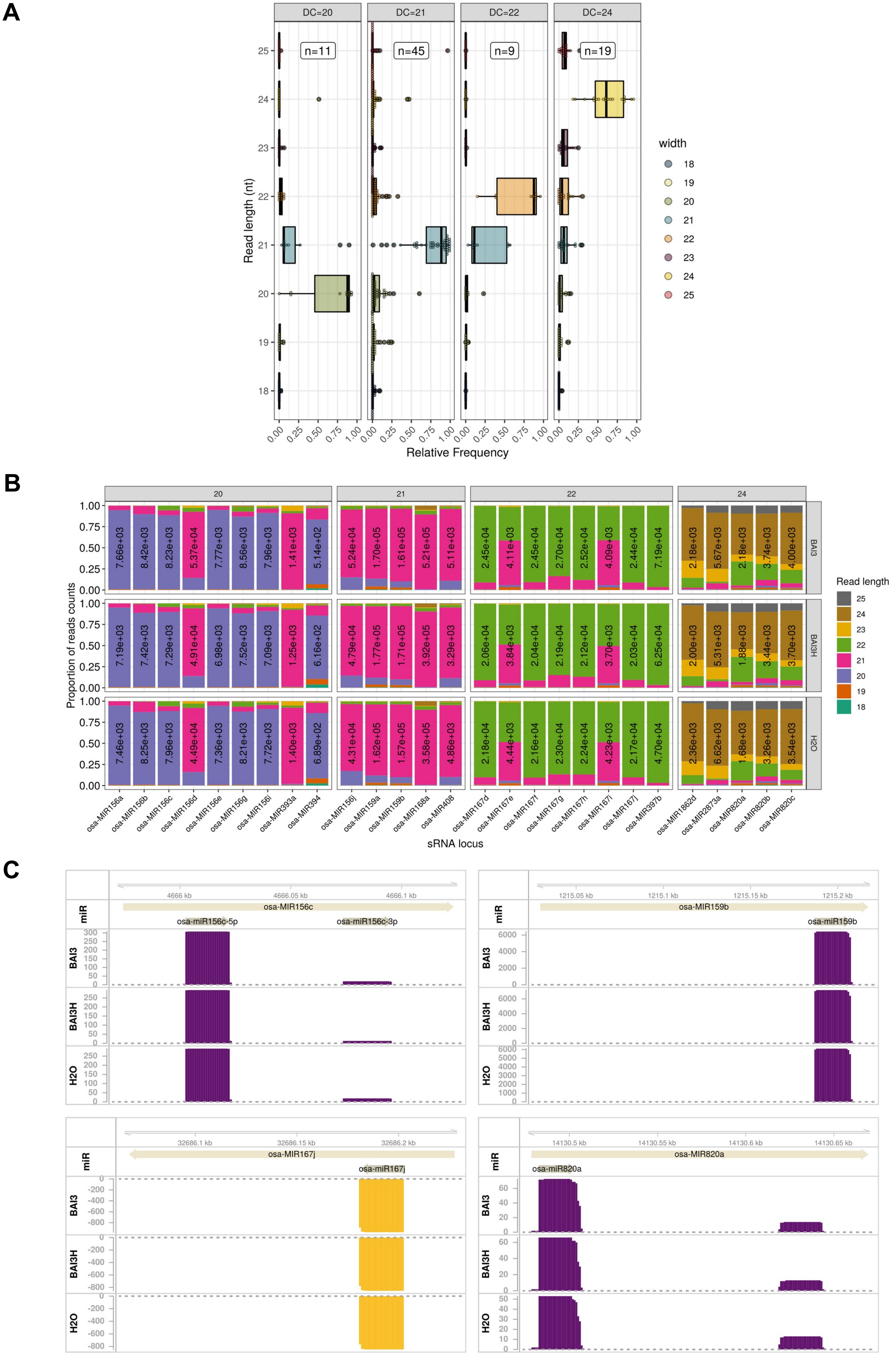
A
DC=20
DC=21
DC=22
DC=24
n=11
n=45
n=9
n=19
Read length (nt)
Relative Frequency
width
18
19
20
21
22
23
24
25
B
Proportion of reads counts
sRNA locus
Read length
BAI3
BAI3H
H2O
osa-MIR156a
osa-MIR156b
osa-MIR156c
osa-MIR156d
osa-MIR156e
osa-MIR156g
osa-MIR156i
osa-MIR393a
osa-MIR394
osa-MIR156j
osa-MIR159a
osa-MIR159b
osa-MIR168a
osa-MIR408
osa-MIR167d
osa-MIR167e
osa-MIR167i
osa-MIR167g
osa-MIR167h
osa-MIR167i
osa-MIR167j
osa-MIR397b
osa-MIR1862d
osa-MIR2873a
osa-MIR820a
osa-MIR820b
osa-MIR820c
C
miR
osa-MIR156c
osa-miR156c-5p
osa-miR156c-3p
osa-MIR159b
osa-miR159b
osa-MIR167j
osa-miR167j
osa-MIR820a
osa-miR820a

Figure S2

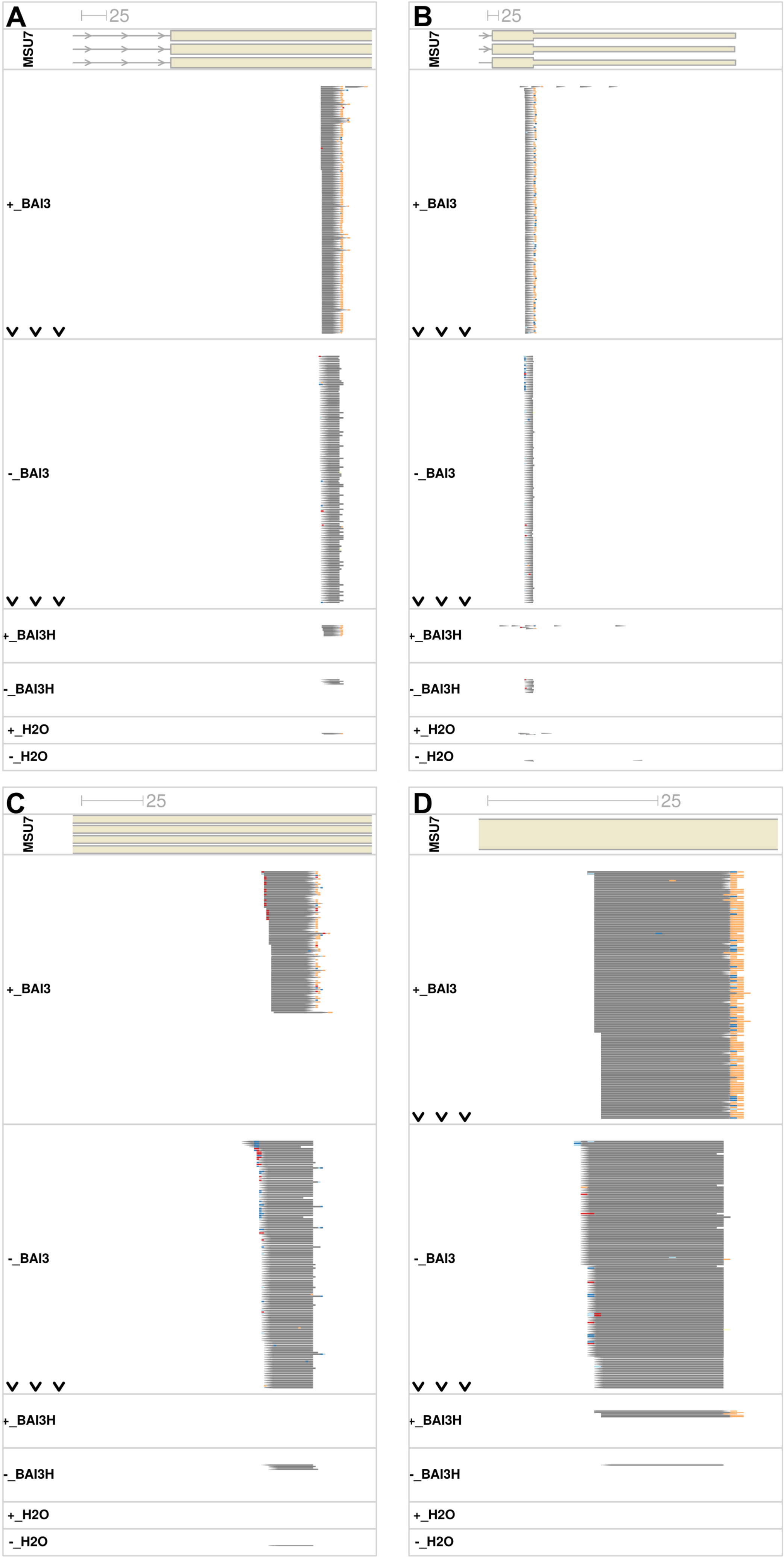

A
25
MSU7
+_BAI3
-_BAI3
+_BAI3H
-_BAI3H
+_H2O
-_H2O
B
25
MSU7
+_BAI3
-_BAI3
+_BAI3H
-_BAI3H
+_H2O
-_H2O
C
25
MSU7
+_BAI3
-_BAI3
+_BAI3H
-_BAI3H
+_H2O
-_H2O
D
25
MSU7
+_BAI3
-_BAI3
+_BAI3H
-_BAI3H
+_H2O
-_H2O

Figure S3

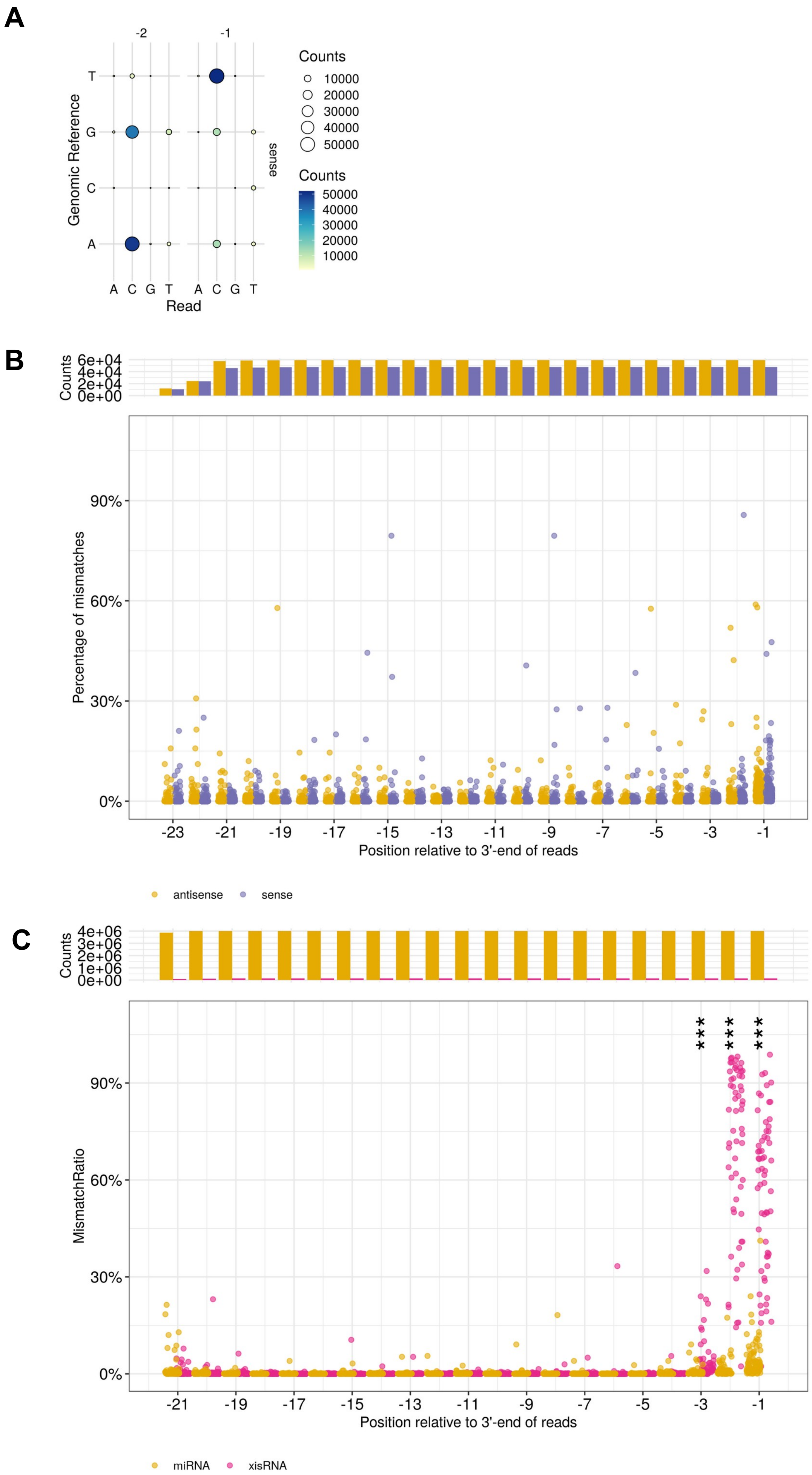
A
-2
-1
Genomic Reference
T
G
C
A
A C G T A C G T
Read
sense
Counts
10000
20000
30000
40000
50000
Counts
50000
40000
30000
20000
10000
B
Counts
6e+04
4e+04
2e+04
0e+00
Percentage of mismatches
90%
60%
30%
0%
-23 -21 -19 -17 -15 -13 -11 -9 -7 -5 -3 -1
Position relative to 3'-end of reads
antisense
sense
C
Counts
4e+06
3e+06
2e+06
1e+06
0e+00
MismatchRatio
90%
60%
30%
0%
-21 -19 -17 -15 -13 -11 -9 -7 -5 -3 -1
Position relative to 3'-end of reads
miRNA
xisRNA

Figure S4

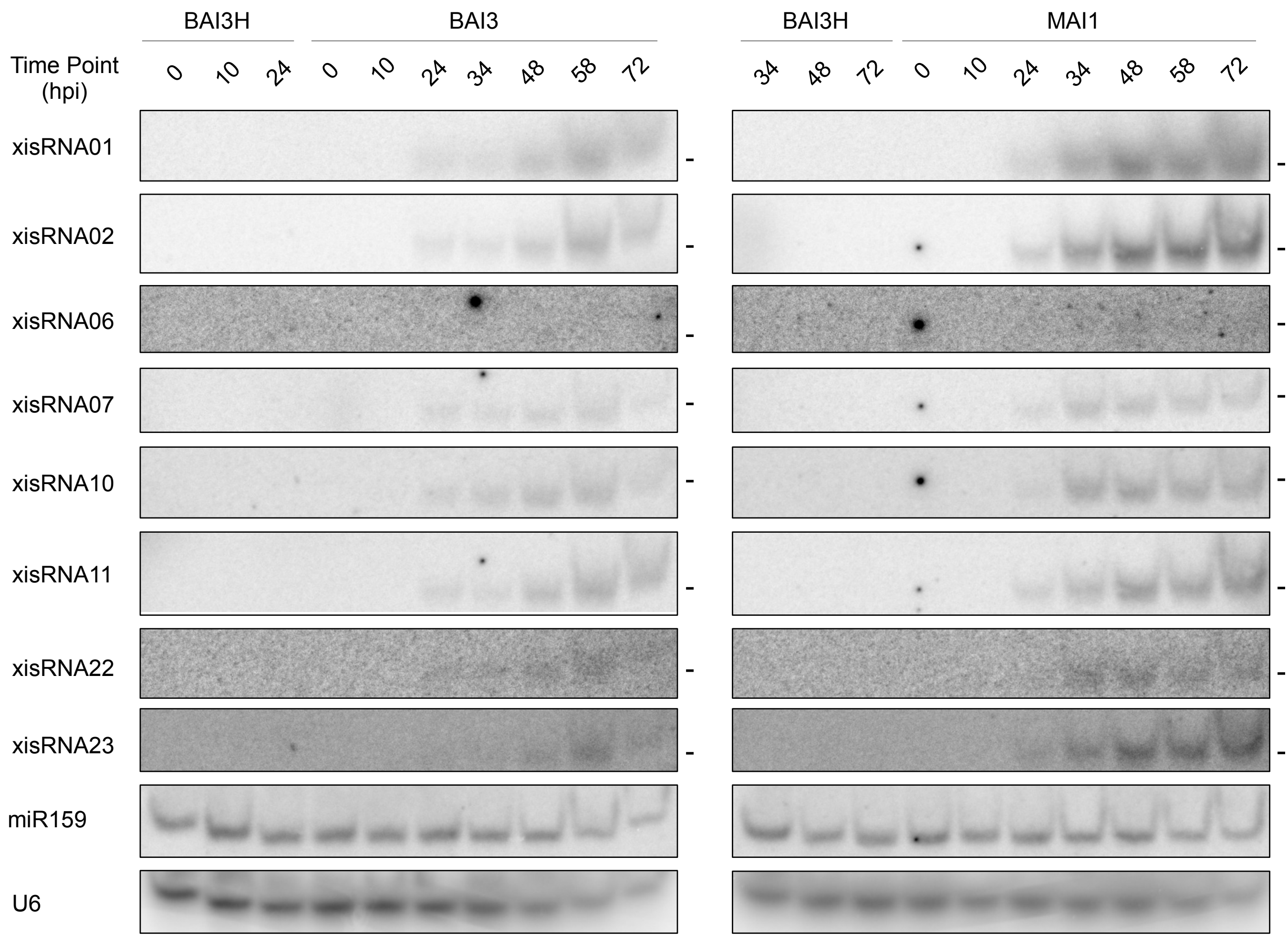
BAI3H
BAI3
BAI3H
MAI1
Time Point (hpi)
0 10 24 0 10 24 34 48 58 72
34 48 72 0 10 24 34 48 58 72
xisRNA01
xisRNA02
xisRNA06
xisRNA07
xisRNA10
xisRNA11
xisRNA22
xisRNA23
miR159
U6

Figure S5

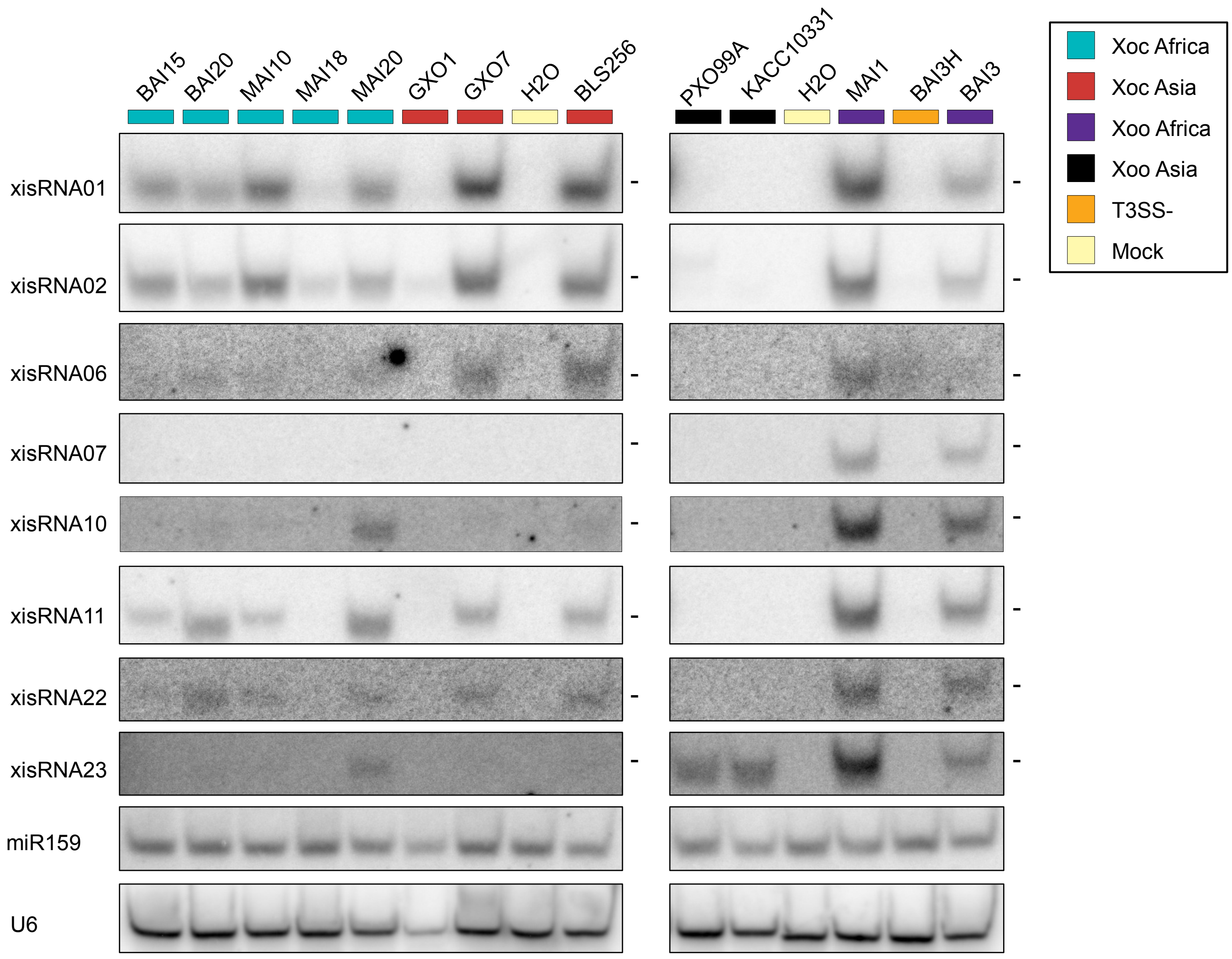
BAI15
BAI20
MAI10
MAI18
MAI20
GXO1
GXO7
H2O
BLS256
PXO99A
KACC10331
H2O
MAI1
BAI3H
BAI3
Xoc Africa
Xoc Asia
Xoo Africa
Xoo Asia
T3SS-
Mock
xisRNA01
xisRNA02
xisRNA06
xisRNA07
xisRNA10
xisRNA11
xisRNA22
xisRNA23
miR159
U6

Figure S6

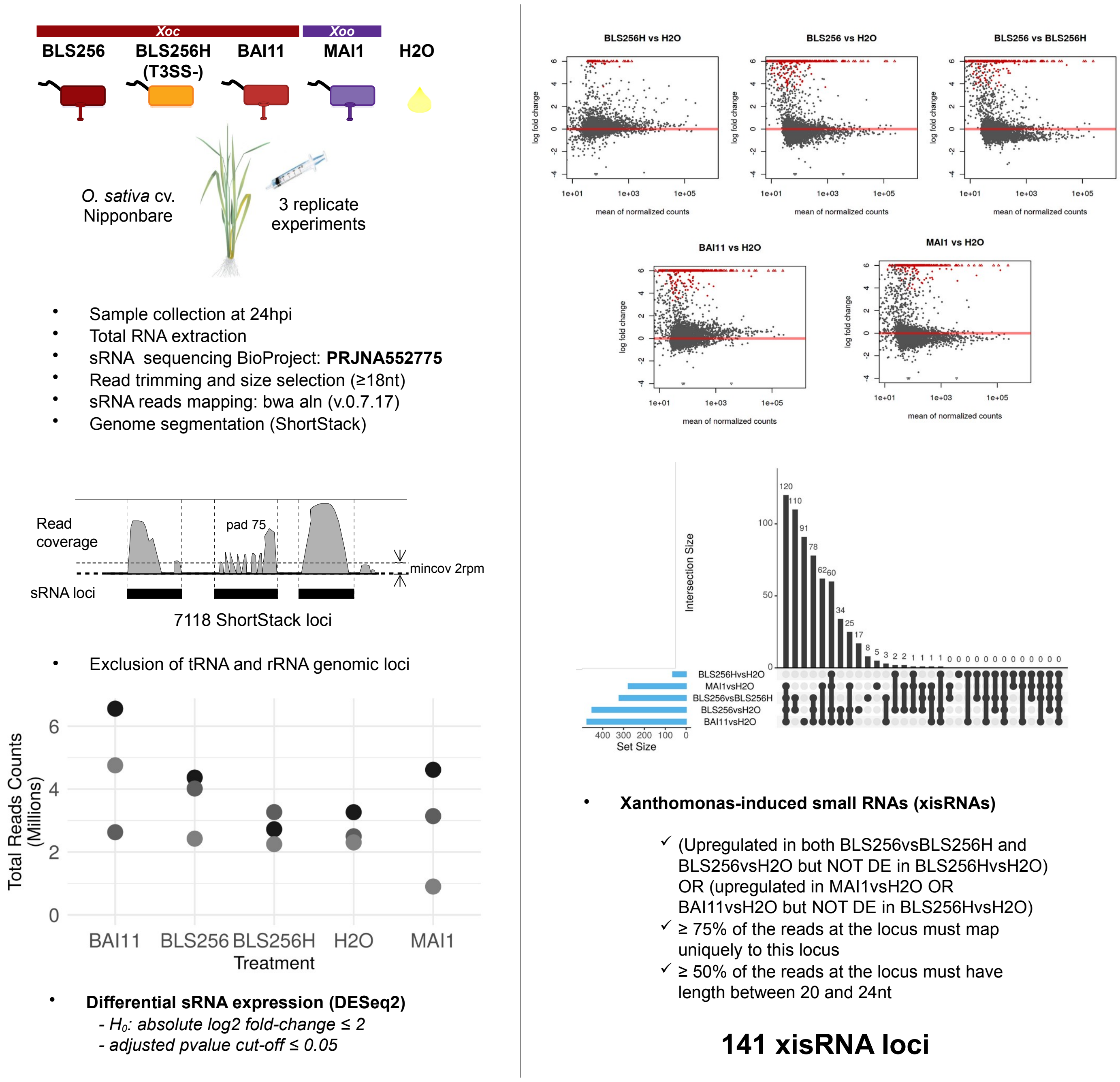
Xoc
Xoo
BLS256
BLS256H
(T3SS-)
BAI11
MAI1
H2O
O. sativa cv.
Nipponbare
3 replicate
experiments
Sample collection at 24hpi
Total RNA extraction
sRNA sequencing BioProject: PRJNA552775
Read trimming and size selection (≥18nt)
sRNA reads mapping: bwa aln (v.0.7.17)
Genome segmentation (ShortStack)
Read
coverage
pad 75
mincov 2rpm
sRNA loci
7118 ShortStack loci
Exclusion of tRNA and rRNA genomic loci
Total Reads Counts
(Millions)
0
2
4
6
BAI11
BLS256
BLS256H
H2O
MAI1
Treatment
Differential sRNA expression (DESeq2)
- H_0: absolute log2 fold-change ≤ 2
- adjusted pvalue cut-off ≤ 0.05
BLS256H vs H2O
BLS256 vs H2O
BLS256 vs BLS256H
BAI11 vs H2O
MAI1 vs H2O
log fold change
mean of normalized counts
1e+01
1e+03
1e+05
Intersection Size
120
110
91
78
62
60
34
25
17
8
5
3 2 2 1 1 1 1 0 0 0 0 0 0 0 0 0 0 0 0
100
50
0
BLS256HvsH2O
MAI1vsH2O
BLS256vsBLS256H
BLS256vsH2O
BAI11vsH2O
400 300 200 100 0
Set Size
Xanthomonas-induced small RNAs (xisRNAs)
(Upregulated in both BLS256vsBLS256H and BLS256vsH2O but NOT DE in BLS256HvsH2O) OR (upregulated in MAI1vsH2O OR BAI11vsH2O but NOT DE in BLS256HvsH2O)
≥ 75% of the reads at the locus must map uniquely to this locus
≥ 50% of the reads at the locus must have length between 20 and 24nt
141 xisRNA loci

Figure S7

A

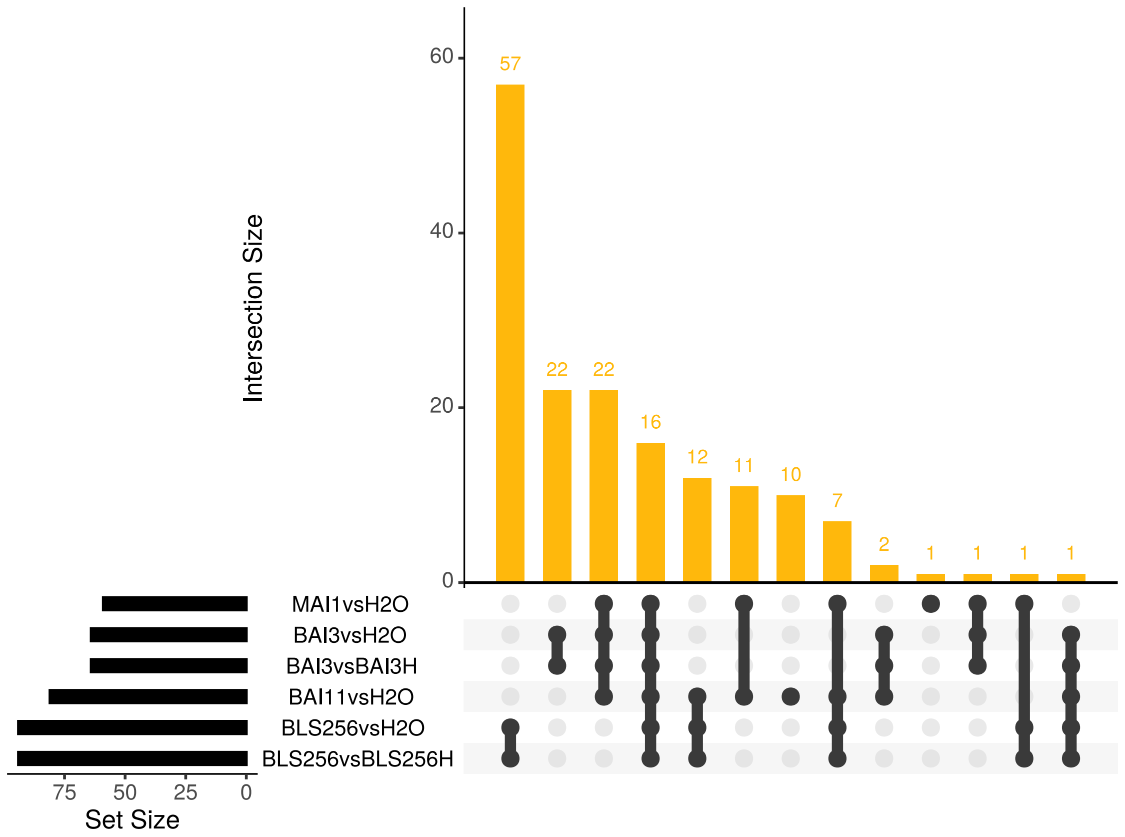

Intersection Size
57
22
22
16
12
11
10
7
2
1
1
1
1
MAI1vsH2O
BAI3vsH2O
BAI3vsBAI3H
BAI11vsH2O
BLS256vsH2O
BLS256vsBLS256H
Set Size

B

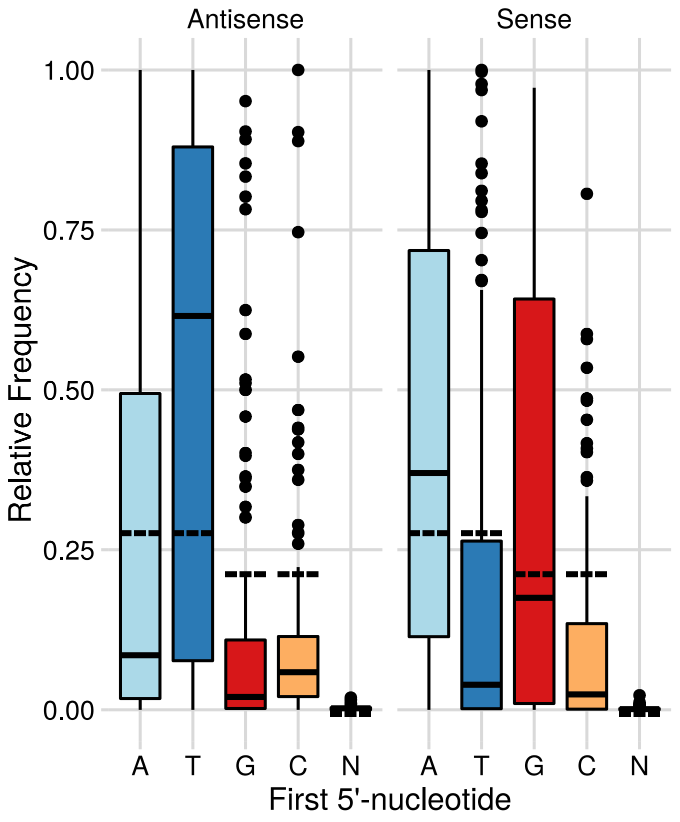

Antisense
Sense
Relative Frequency
A T G C N A T G C N
First 5'-nucleotide

Figure S8

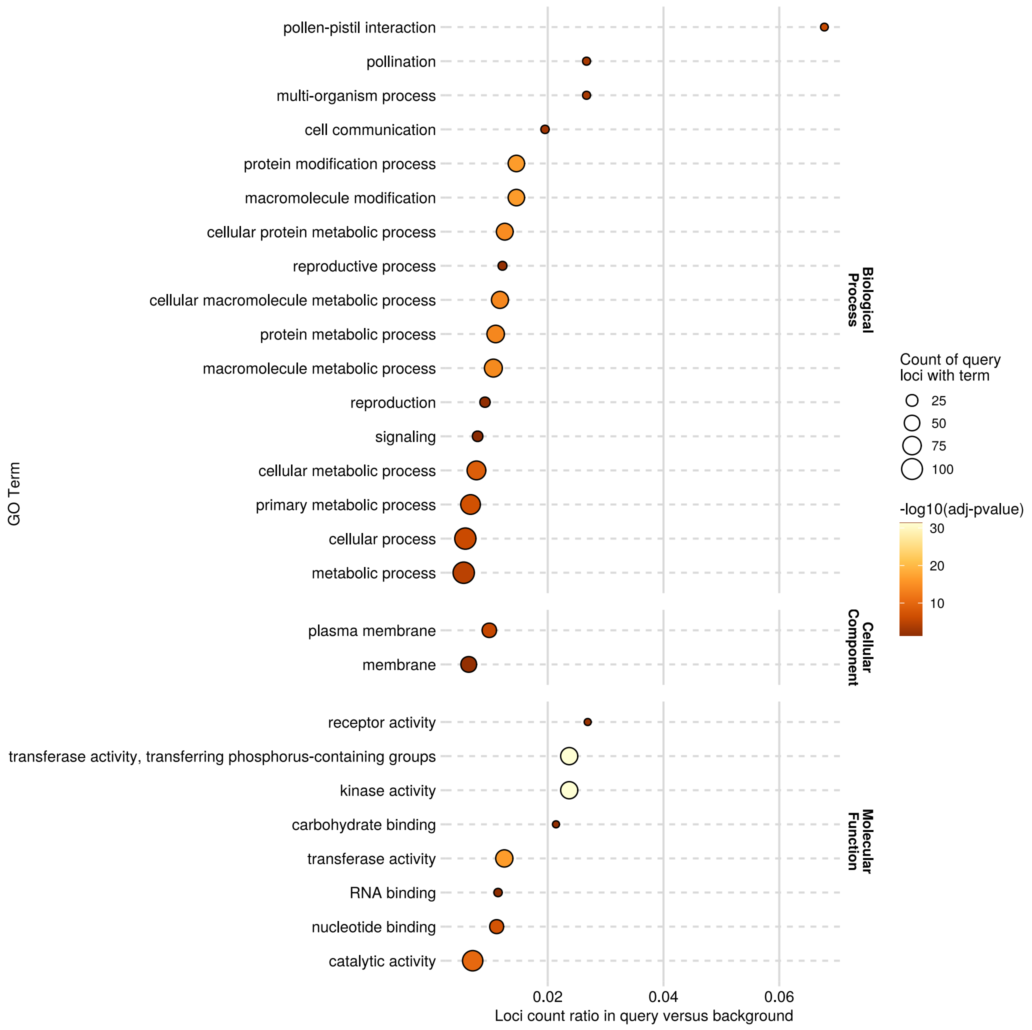
pollen-pistil interaction
pollination
multi-organism process
cell communication
protein modification process
macromolecule modification
cellular protein metabolic process
reproductive process
cellular macromolecule metabolic process
protein metabolic process
macromolecule metabolic process
reproduction
signaling
cellular metabolic process
primary metabolic process
cellular process
metabolic process
Biological Process
plasma membrane
membrane
Cellular Component
receptor activity
transferase activity, transferring phosphorus-containing groups
kinase activity
carbohydrate binding
transferase activity
RNA binding
nucleotide binding
catalytic activity
Molecular Function
GO Term
0.02
0.04
0.06
Loci count ratio in query versus background
Count of query loci with term
25
50
75
100
-log10(adj-pvalue)
30
20
10

Figure S9

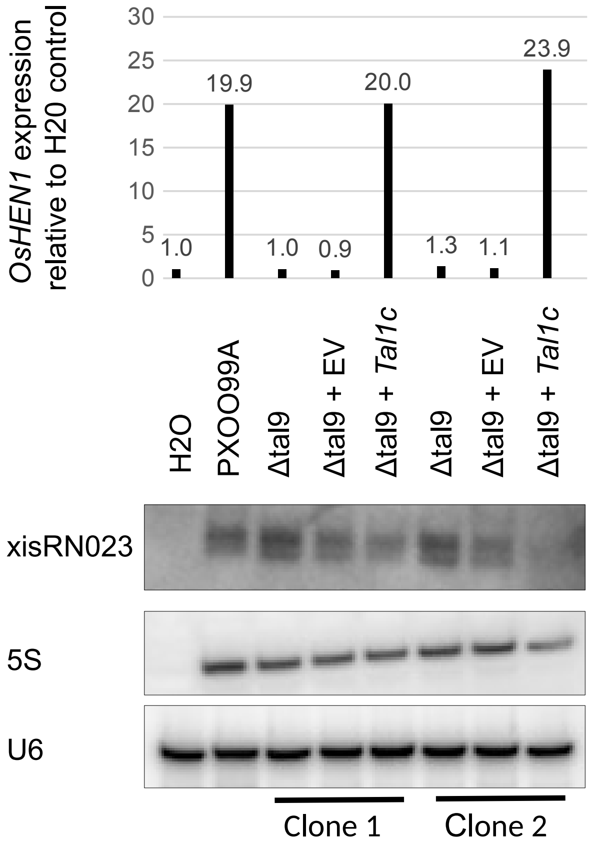

OsHEN1 expression relative to H20 control
30
25
20
15
10
5
0
1.0
19.9
1.0
0.9
20.0
1.3
1.1
23.9
H2O
PXOO99A
Δtal9
Δtal9 + EV
Δtal9 + *Tal1c*
Δtal9
Δtal9 + EV
Δtal9 + *Tal1c*
xisRN023
5S
U6
Clone 1
Clone 2

Figure S10

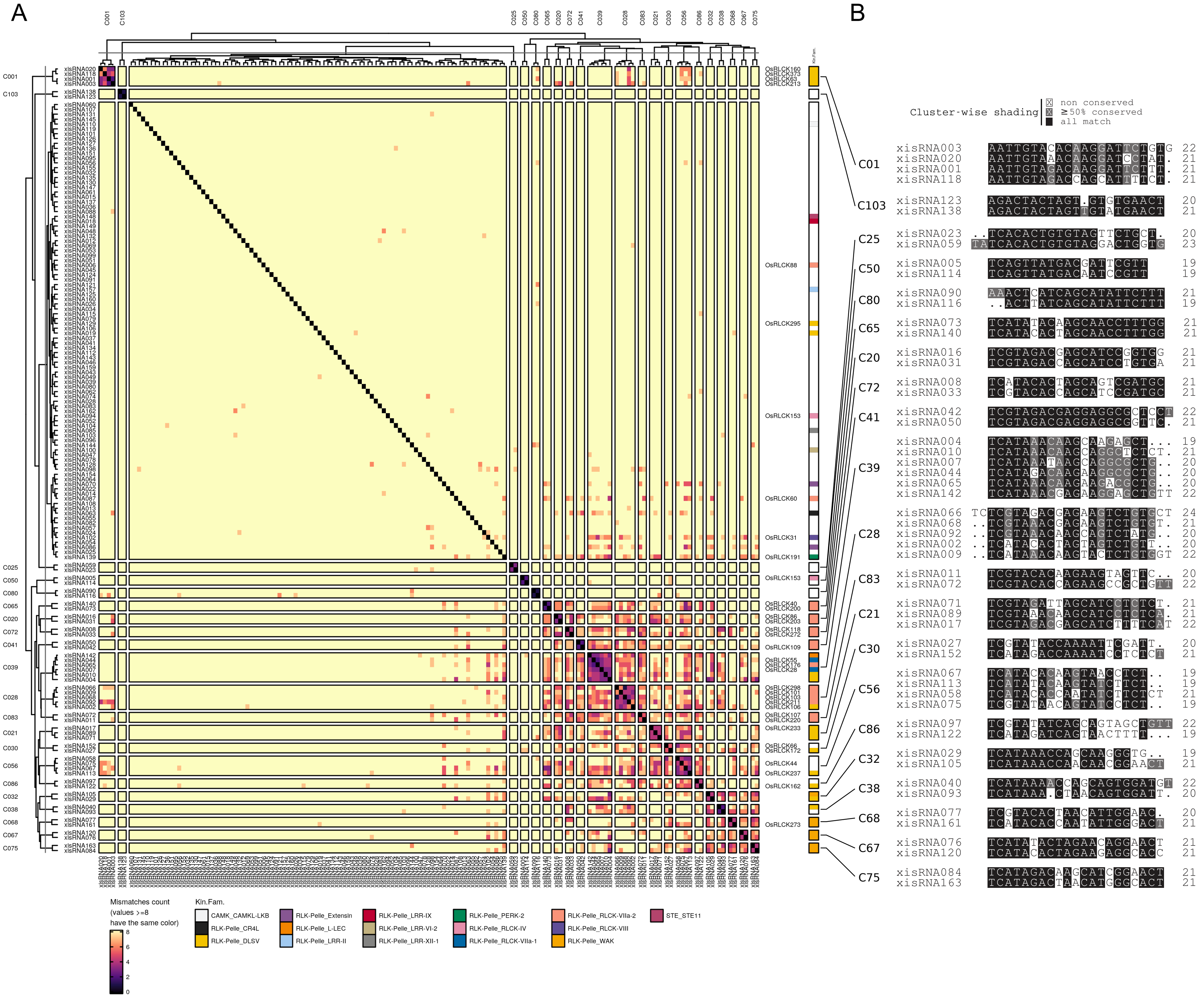
A
B
Mismatches count
(values >=8
have the same color)
8
6
4
2
0
Kin.Fam.
CAMK_CAMKL-LKB
RLK-Pelle_CR4L
RLK-Pelle_DLSV
RLK-Pelle_Extensin
RLK-Pelle_L-LEC
RLK-Pelle_LRR-II
RLK-Pelle_LRR-IX
RLK-Pelle_LRR-VI-2
RLK-Pelle_LRR-XII-1
RLK-Pelle_PERK-2
RLK-Pelle_RLCK-IV
RLK-Pelle_RLCK-VIIa-1
RLK-Pelle_RLCK-VIIa-2
RLK-Pelle_RLCK-VIII
RLK-Pelle_WAK
STE_STE11
Cluster-wise shading
non conserved
≥50% conserved
all match
C01
C103
C25
C50
C80
C65
C20
C72
C41
C39
C28
C83
C21
C30
C56
C86
C32
C38
C68
C67
C75

Figure S11

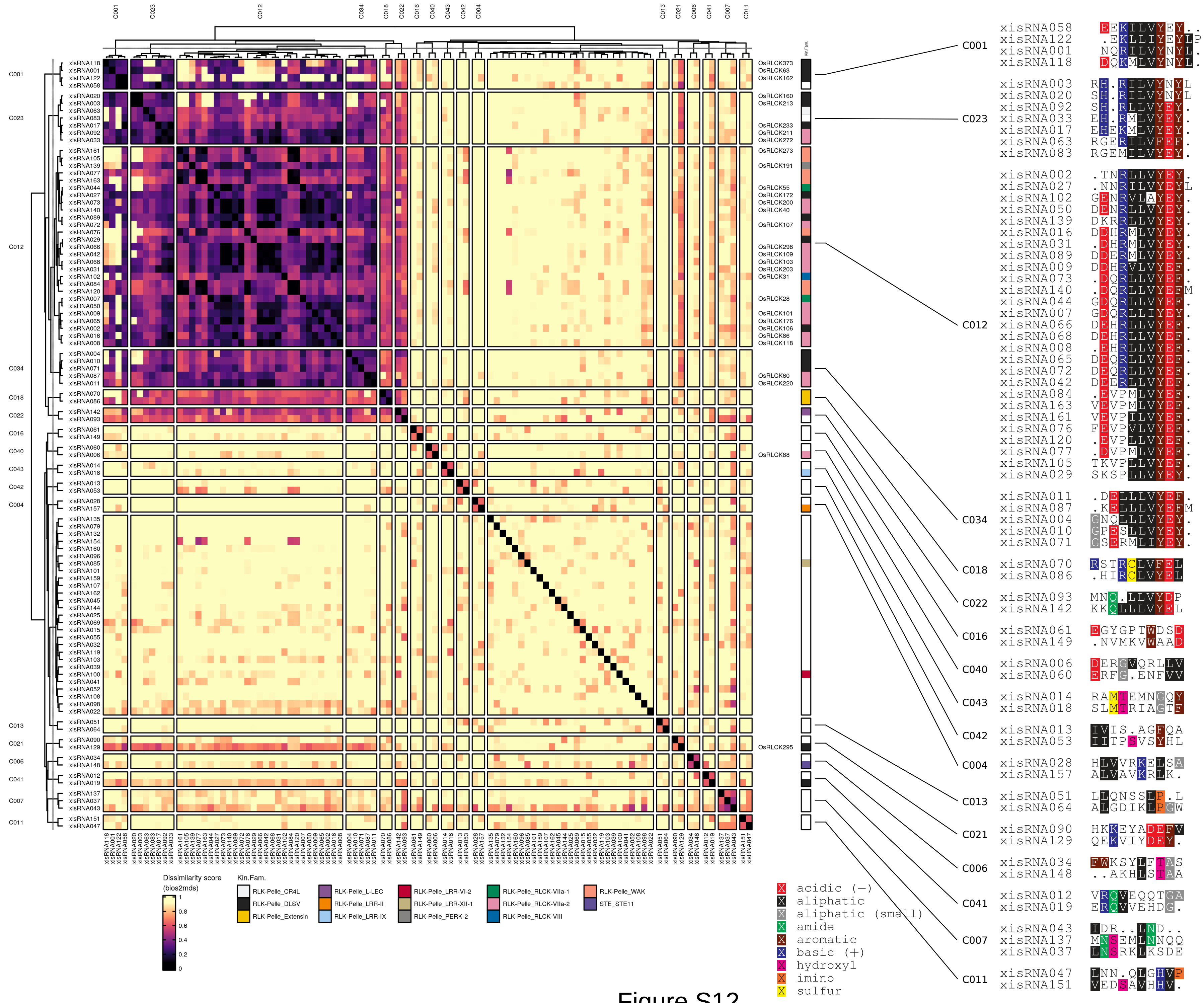
C001
C023
C012
C034
C018
C022
C016
C040
C043
C042
C004
C013
C021
C006
C041
C007
C011
Dissimilarity score
(bios2mds)
Kin.Fam.
RLK-Pelle_CR4L
RLK-Pelle_DLSV
RLK-Pelle_Extensin
RLK-Pelle_L-LEC
RLK-Pelle_LRR-II
RLK-Pelle_LRR-IX
RLK-Pelle_LRR-VI-2
RLK-Pelle_LRR-XII-1
RLK-Pelle_PERK-2
RLK-Pelle_RLCK-VIIa-1
RLK-Pelle_RLCK-VIIa-2
RLK-Pelle_RLCK-VIII
RLK-Pelle_WAK
STE_STE11
acidic (−)
aliphatic
aliphatic (small)
amide
aromatic
basic (+)
hydroxyl
imino
sulfur

Figure S12

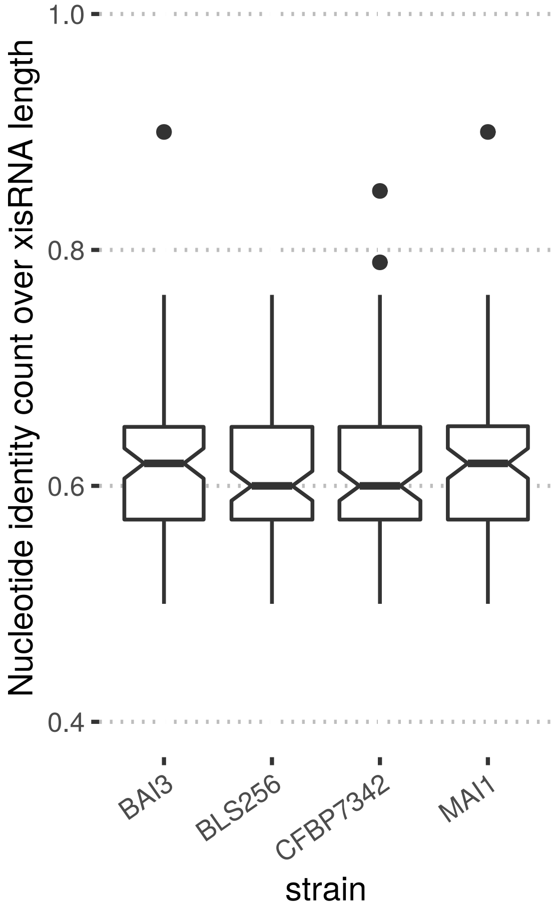

Nucleotide identity count over xisRNA length
1.0
0.8
0.6
0.4
BAI3
BLS256
CFBP7342
MAI1
strain

Figure S13

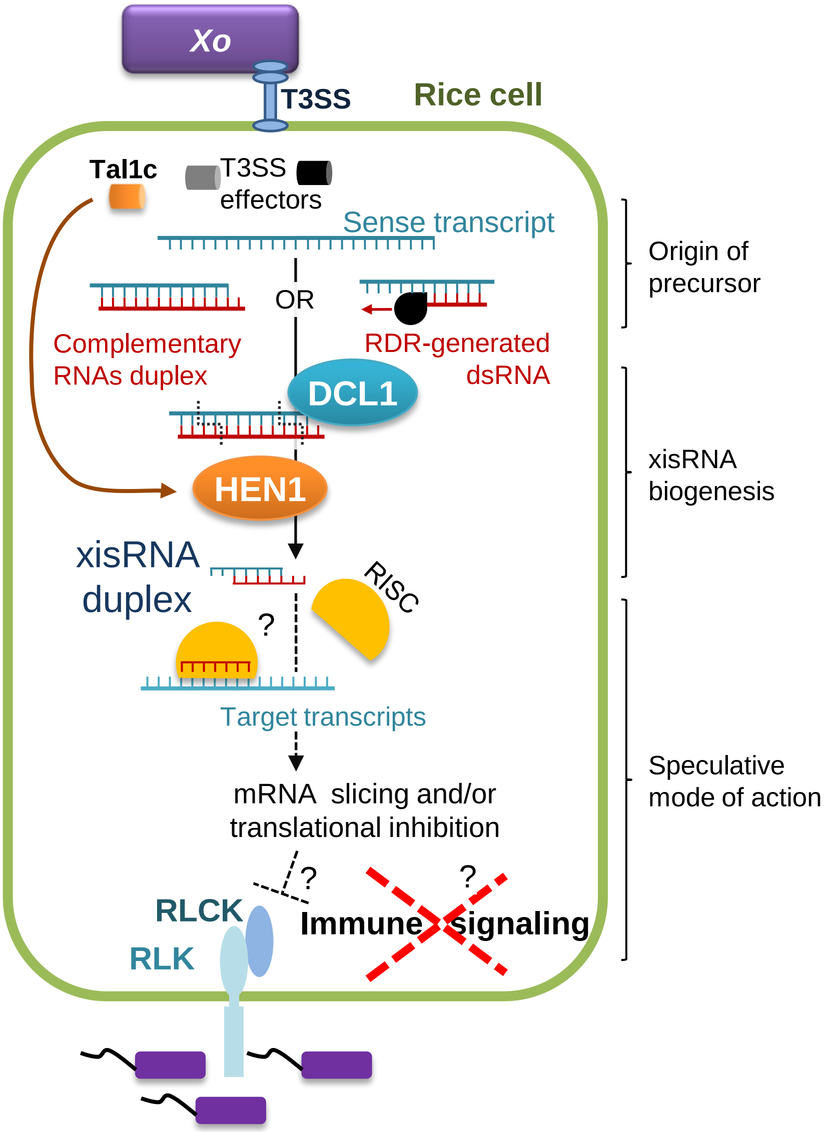
Xo
T3SS
Rice cell
Tal1c
T3SS
effectors
Sense transcript
OR
Complementary
RNAs duplex
RDR-generated
dsRNA
DCL1
HEN1
xisRNA
duplex
RISC
?
Target transcripts
mRNA slicing and/or
translational inhibition
?
?
RLCK
RLK
Immune signaling
Origin of
precursor
xisRNA
biogenesis
Speculative
mode of action

Figure S14

Supplementary Figure S15

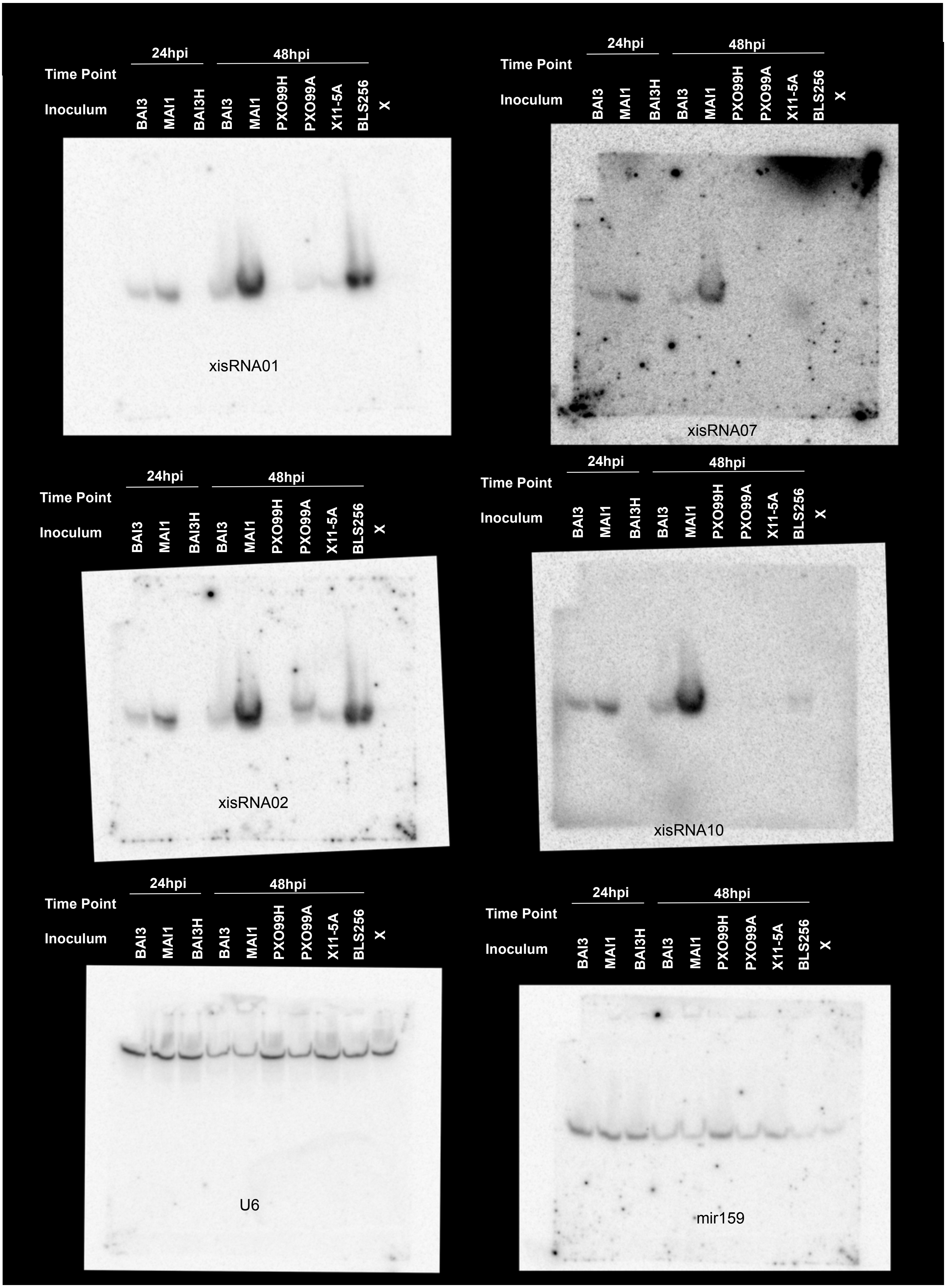

Uncropped images scans for Figure 2A

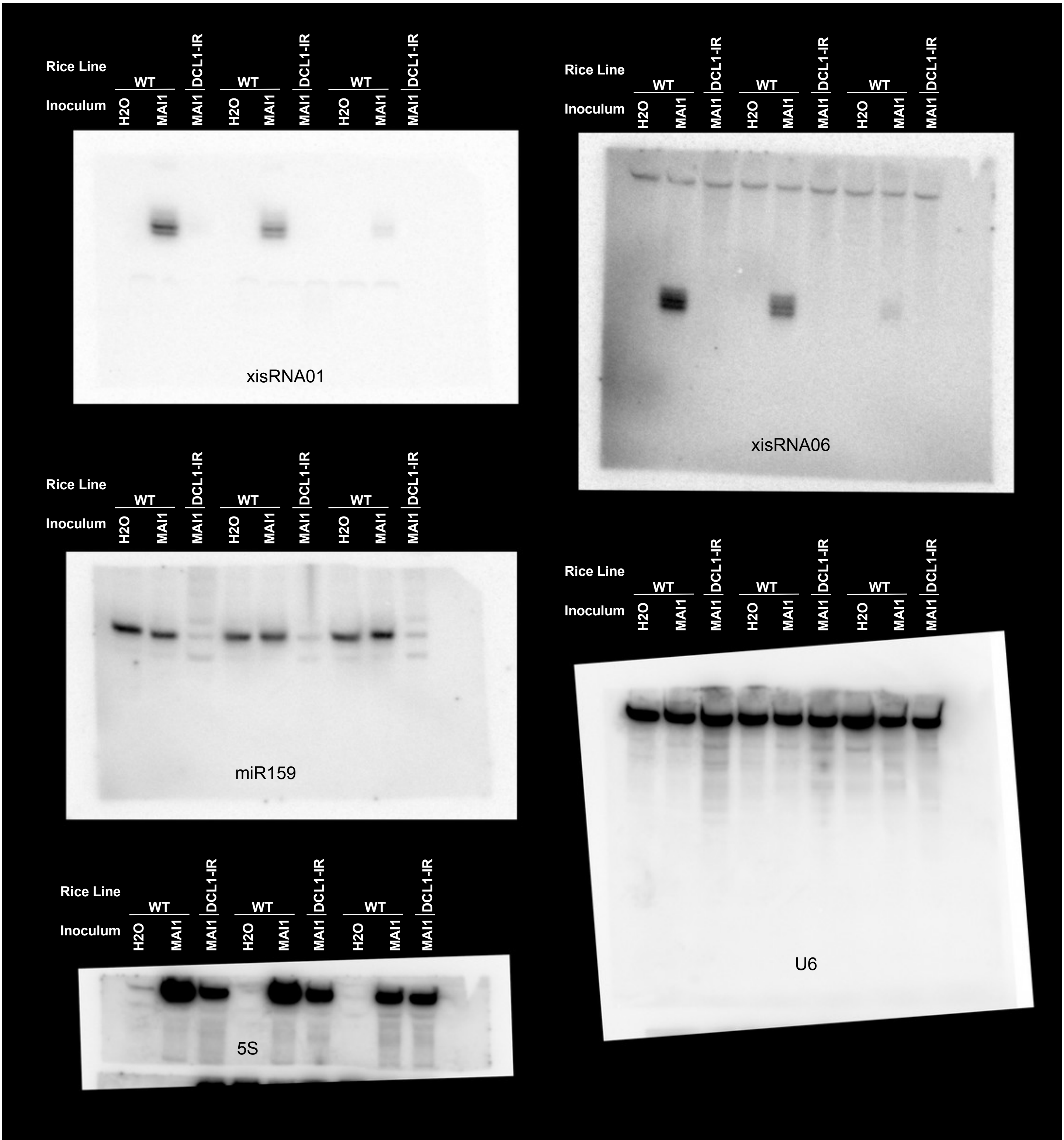

Uncropped images scans for Figure 5A

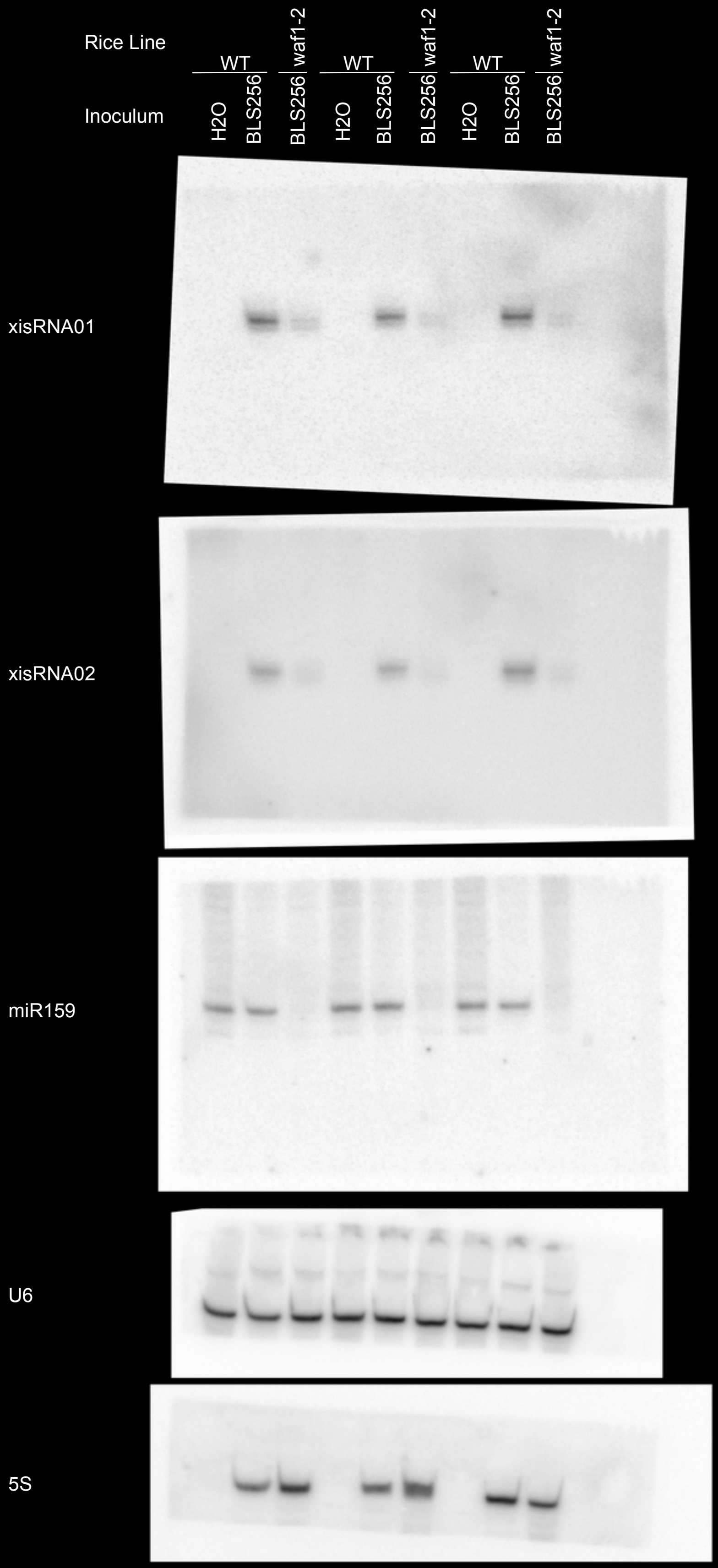

Uncropped images scans for Figure 5B

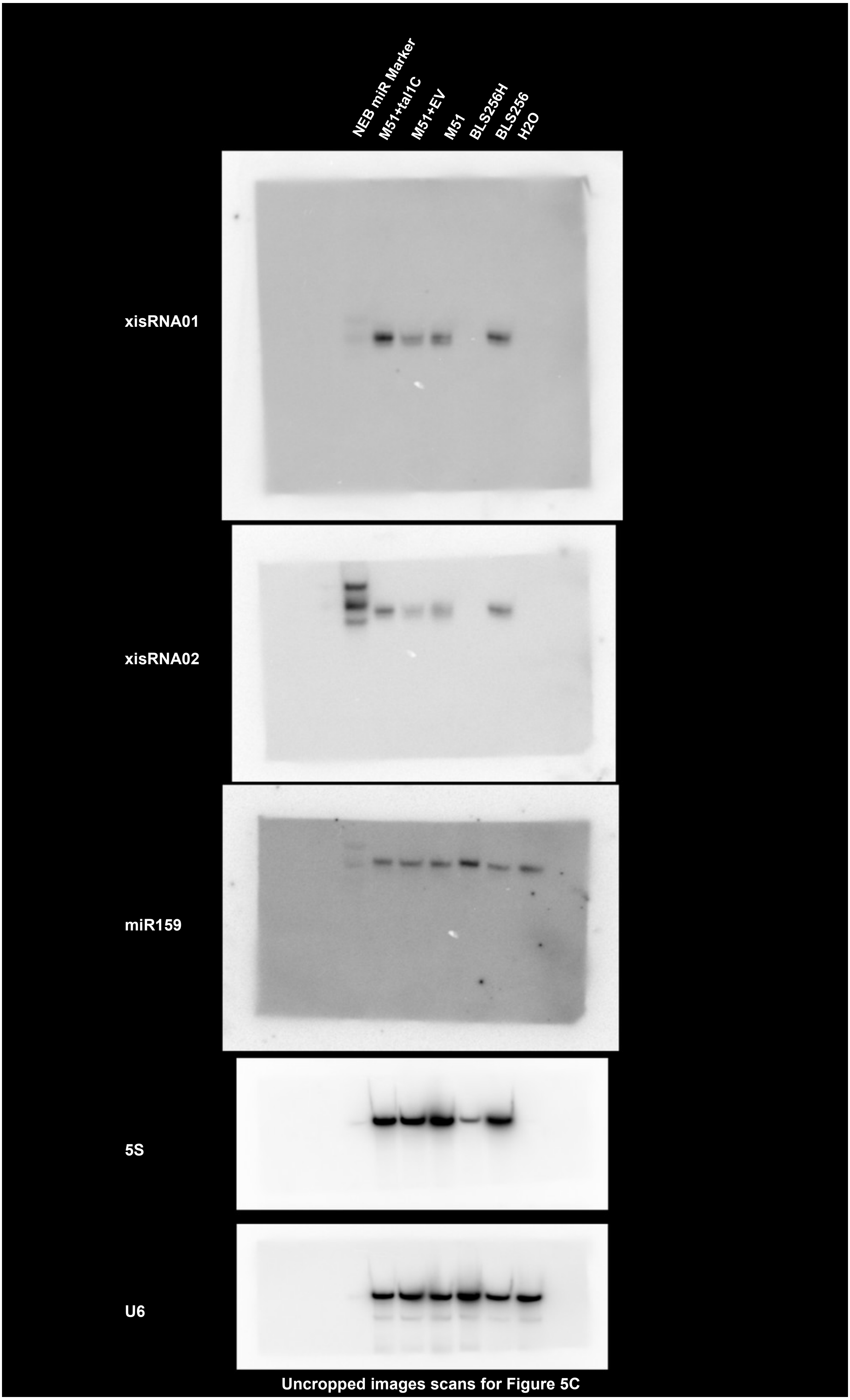

Uncropped images scans for Figure 5C

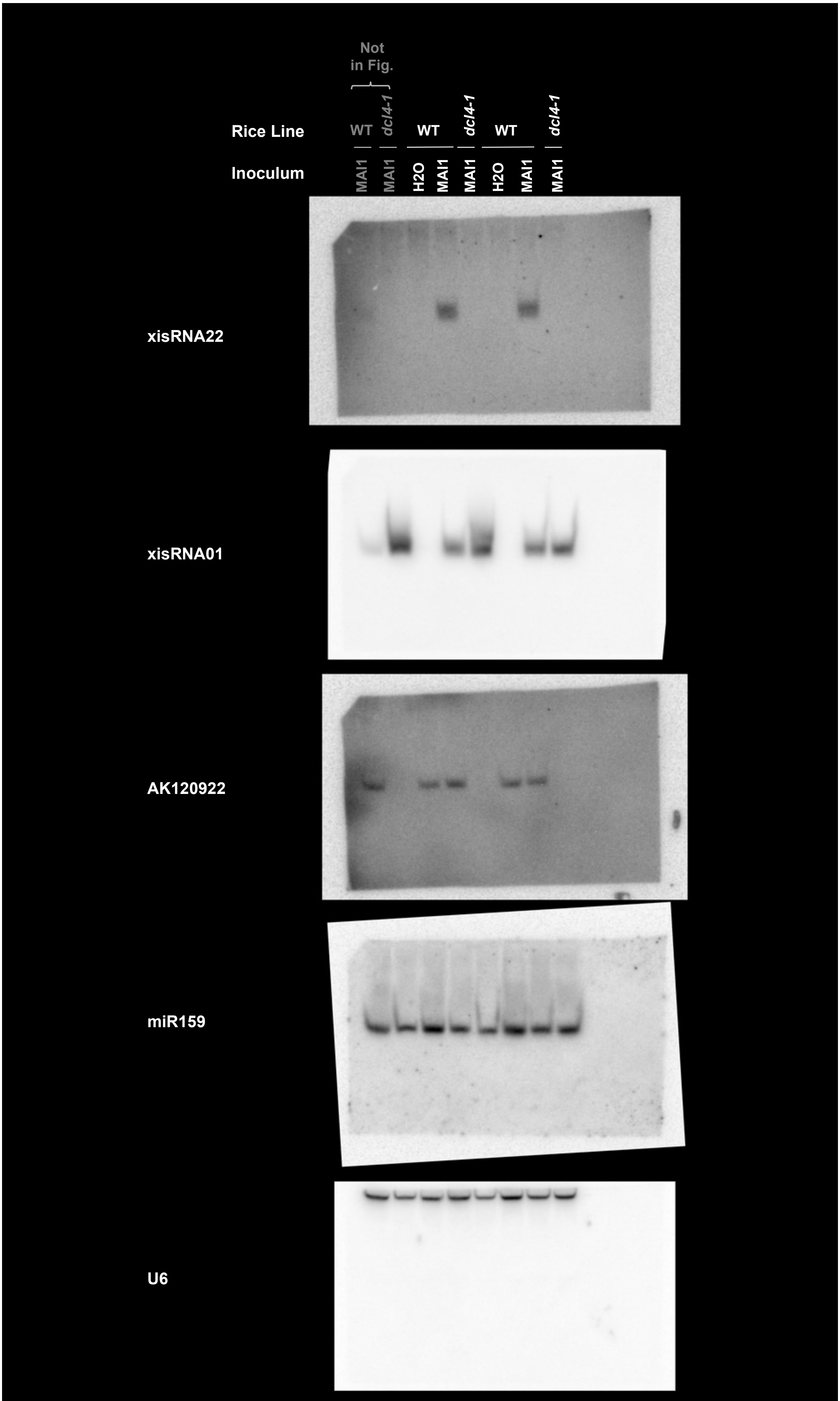

Uncropped images scans for Figure 5E

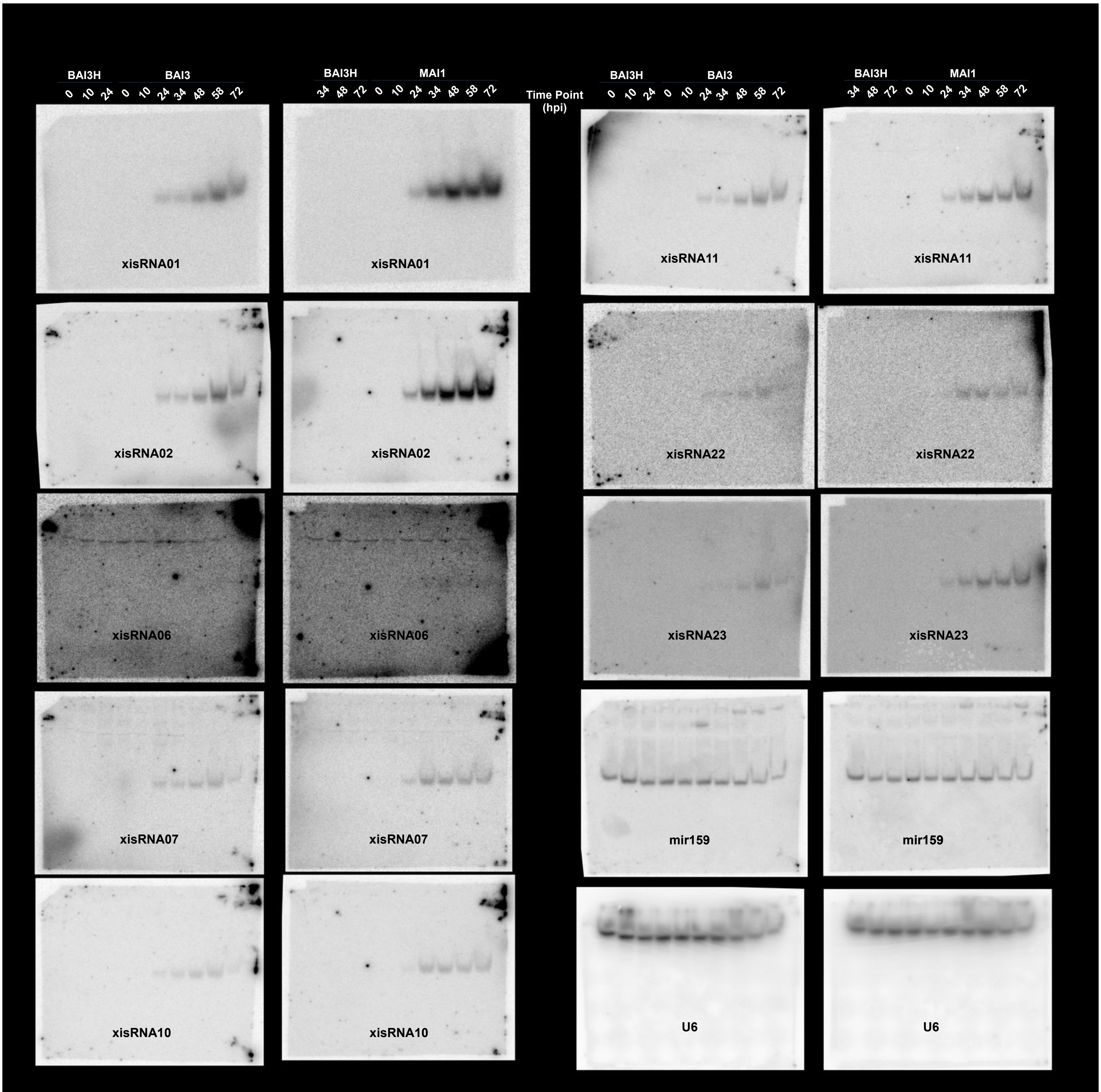

Uncropped images scans for Supplementary Figure S5

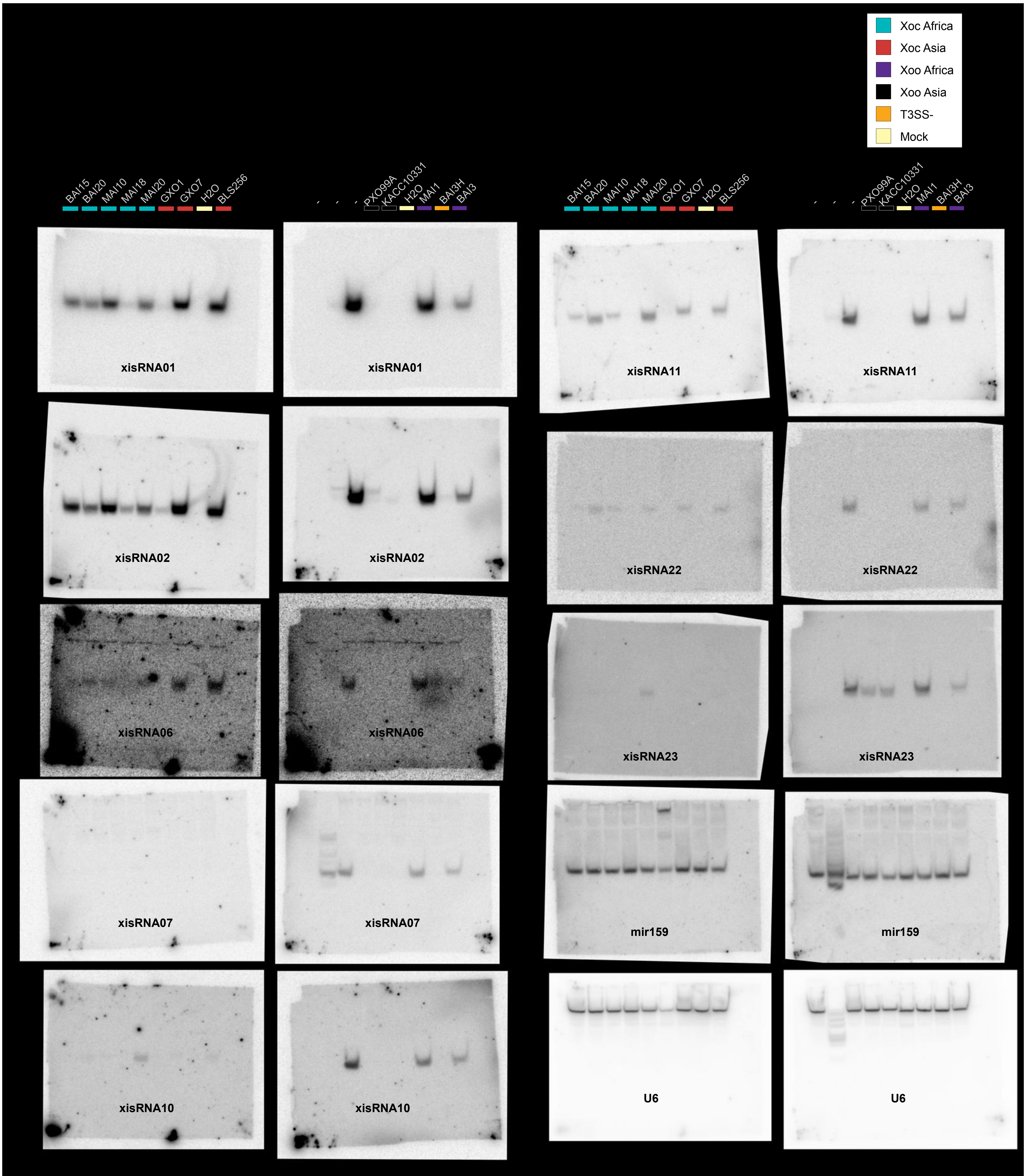

Uncropped images scans for Supplementary Figure S6

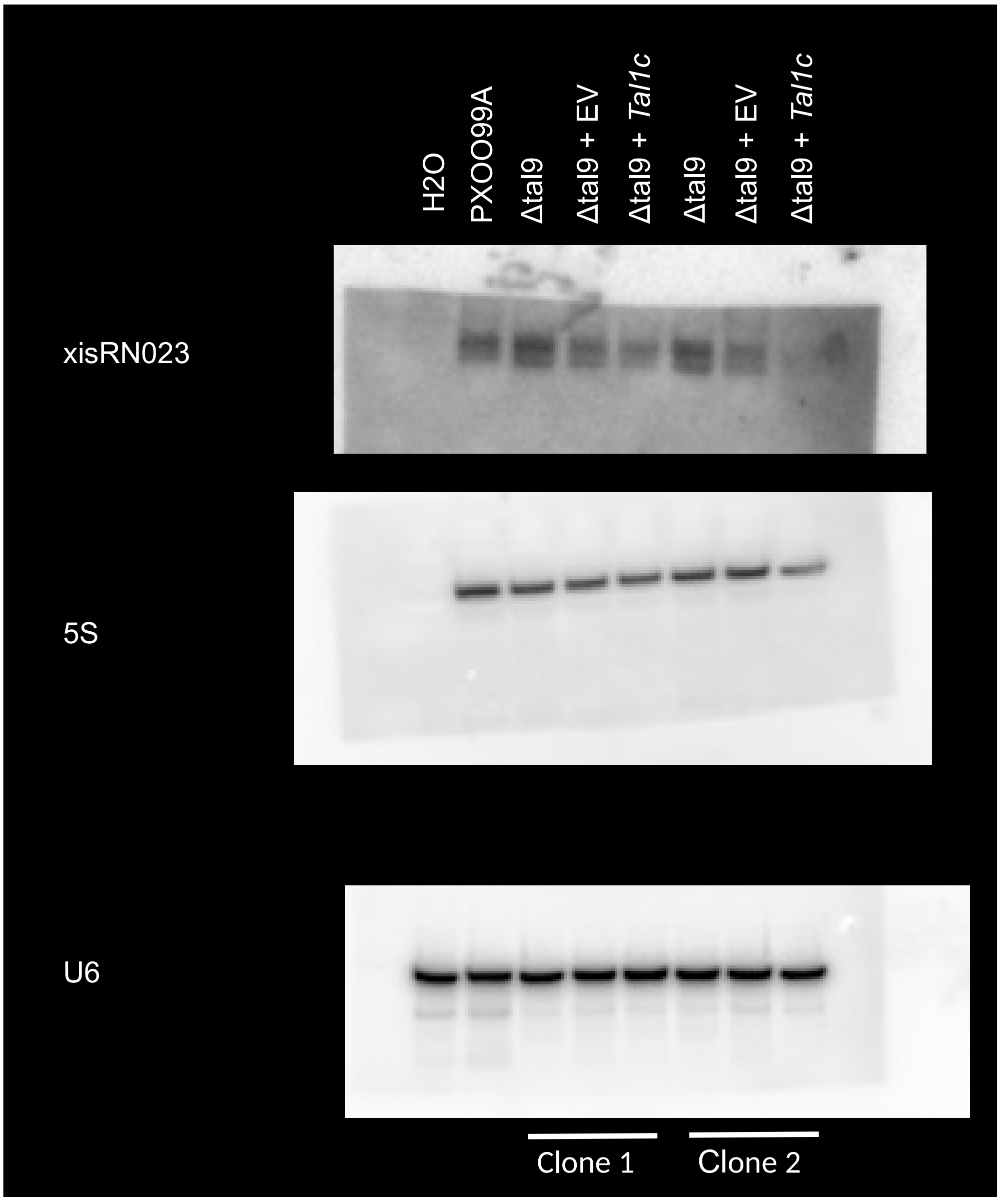

Uncropped images scans for Supplementary Figure S10

Supplementary Table S1: List of the differentially expressed experimental sRNAs loci in the BAI3 dataset.
A short description of the content of the columns is provided as follows:
seqnames: rice chromosome name
start: starting position on chromosome
end: end position on chromosome
width: span of the loci in bp
strand: sRNA locus strand as defined by ShortStack
gbrowse_location: genomic location of the loci formated for easy copy and paste in a genome browser.
Name: label of the loci
For the “FracTop”, “MajorRNA”, “MajorRNAReads”, “Complexity”, “DicerCall”, “MIRNA”and “PhaseScore” columns, please refer to the ShortStack manual for a description of these values.
OlapGene_Names: MSU identifier of sRNA loci overlapping annotation elements
OlapGene_Note: MSU description of sRNA loci overlapping annotation elements
OlapSimpleTrx_Names: Identifier of the MSU locus if the xiRNA locus overlap with an annotated transcript.
DEpattern_BAI3vsH2O_BAI3vsBAI3H_BAI3HvsH2O: binary string encoding the differential expression call in the comparisons listed in the column name; 1 indicates that the sRNA locus was designated as differentially expressed.
%01_Hit.s.: percentage of reads that map to a single best location on the rice genome
%02_Hit.s.: percentage of reads that map to two locations with maximal best scores on the rice genome
%20_nt: percentage of reads of size 20nt
%21_nt: percentage of reads of size 21nt
%22_nt: percentage of reads of size 22nt
%23_nt: percentage of reads of size 23nt
%24_nt: percentage of reads of size 24nt
EditDistance_00: percentage of reads with an edit distance equal to 0 relative to the genome sequence
EditDistance_01: percentage of reads with an edit distance equal to 1 relative to the genome sequence
EditDistance_02: percentage of reads with an edit distance equal to 2 relative to the genome sequence
pri_miRNA: name of overlapping pri-miRNA locus
xisRNAName: name of the corresponding xisRNA
rpm_BAI3: average expression in rpm in the BAI3 treatment
rpm_BAI3H: average expression in rpm in the BAI3H treatment
rpm_H2O: average expression in rpm in the H2O treatment
Column names with a “de_” prefix correspond to the DESeq2 output. Please refer to the DESeq2 manual for a description of these values.

Supplementary Table S2: List of the differentially expressed experimental sRNAs loci in the Diversity dataset.
See the legend of Supplementary Table S1

Supplementary Table S3: xisRNAs features.
Column names are self-explanatory and refers to the values depicted graphically in Figure 3.

Supplementary Table S4: *X. oryzae* strains used in this study.

Strain Designation	Other Designation	Description	References
BAI3	BAI3^R	A rifampicin resistant and fully virulent derivative of the BAI3 wild type *Xoo* strain from Burkina Faso	(2)
BAI3H	BAI3^{R}ΔhrcC	A T3SS minus mutant derivative of the BAI3 strain	(2)
BAI11		Wild type *Xoc* isolate from Burkina Faso (Itenga)	(3, 4)
BAI15		Wild type *Xoc* isolate from Burkina Faso (Bagré)	(3)
BAI20		Wild type *Xoc* isolate from Burkina Faso (Cascades)	(3)
BLS256		Wild type Philippine Xoc isolate	(5)
BLS256H		A T3SS minus mutant derivative of BLS256	(6)
GXO1		Wild type *Xoc* isolate from China (Guangxi)	(7)
GXO7		Wild type *Xoc* isolate from China (Guangxi)	(7)
KACC10331		Wild type *Xoo* isolate from Korea	(8)
M51		Single marker exchange mutant in the *tal1c* gene of BLS256	(9)
M51 + tal1c		M51 harboring the pKEB31-*tal1c* (pCS472) plasmid which is a pKEB31 containing the *tal1c* gene of *Xoc* BLS256	(10) for the plasmid and this study for the strain.
M51 + EV		M51 harboring the pKEB31-*talΔCRR* (pAC99) plasmid which is a pKEB31 containing the *tal1c* gene of *Xoc* BLS256 deprived of the central repeat domain coding sequence	(10) for the plasmid and this study for the strain.
MAI1		Wild type Xoo isolate from Mali (Niono)	(11)
MAI10		Wild type *Xoc* isolate from Mali (Niono)	(11)
MAI18		Wild type *Xoc* isolate from Mali (Office du Niger)	(3)
MAI20		Wild type *Xoc* isolate from Mali (Office du Niger)	(3)
PXO99A		5-Azacytidine resistant derivative of the race 6 Filipino strain PXO99	(12)
PXO99H	PXO99A ME7	A T3SS defective mutant derivative of the PXO99A strain	(13)
X11-5A		*Xo*-like strain isolated in the USA	(14)

Supplementary Table S5: Oligonucleotide used in this study.

Primer sequence (5'->3')	Target	Reference	Use
AACCAAAGAATCCTTGTCTACAATTATCTT	xisRNA001	This study	LMW northern blots
TAACAGACTACTAGTGTATGAATA	xisRNA002	This study	LMW northern blots
GGGGCGTACAGCGTCTCCTAG	xisRNA006	This study	LMW northern blots
TCAGCGCCTGCTTATTTATGA	xisRNA007	This study	LMW northern blots
AGAGAGCCTGCTTGTTTATGA	xisRNA010	This study	LMW northern blots
TGACGAACTACTTCTTGTGTACGAGTTC	xisRNA011	This study	LMW northern blots
AGTGATATTTGTTTGTCCAGAGATA	xisRNA022	This study	LMW northern blots
CAGCAGAACTACACAGTGTGAGGAAC	xisRNA023	This study	LMW northern blots
CCTGAAAGTTCATGAAGCGGT	AK120922	This study	LMW northern blots
TGTATCGTTCCAATTTTATCGGATGT	U6 snRNA	This study	LMW northern blots
CCTGGCGATGACCTACTCTC	*X. oryzae* 5S ribosomal RNA	This study	LMW northern blots
CAGAGCTCCCTTCAATCCAAA	miR159	This study	LMW northern blots
TCTCGGAAGGGTCTCACACGA	*OsHEN1*	This study	Q-RT-PCR
AGAGCACAGCCGTTGGAACAC	*OsHEN1*	This study	Q-RT-PCR
GAAGTCTCATCCTACCTGAAGAAG	*OsEF-1*α	(15)	Q-RT-PCR
GTCAAGAGCCTCAAGCAAGG	*OsEF-1*α		Q-RT-PCR
CAGACGGTGTATGTAACACC	*OsDCL4* and *dcl4-1* forward	(16)	*dcl4-1* genotyping
CGGACTCCAAGACGCAATATGT	*OsDCL4* reverse	(16)	*dcl4-1* genotyping
TTGCGATCACCACAGCTTGC	*dcl4-1* reverse	(16)	*dcl4-1* genotyping
AAGGAGAGACTCAGAGCTTC	*OsHEN1* forward	(17) and Itoh Jun-Ichi personnal communication	*waf1-2* CAPS marker (EcoRV cleavage is diagnostic of the *waf1-2* allele)
ATGAGAACAGAAGGTGTGCC	*OsHEN1* reverse		*waf1-2* CAPS marker (EcoRV cleavage is diagnostic of the *waf1-2* allele)

SUPPLEMENTARY REFERENCES

1. Lunardon,A., Johnson,N.R., Hagerott,E., Phifer,T., Polydore,S., Coruh,C. and Axtell,M.J. (2020) Integrated annotations and analyses of small RNA-producing loci from 47 diverse plants. *Genome Res*, **30**, 497–513.

2. Yu,Y., Streubel,J., Balzergue,S., Champion,A., Boch,J., Koebnik,R., Feng,J., Verdier,V. and Szurek,B. (2011) Colonization of rice leaf blades by an African strain of Xanthomonas oryzae pv. oryzae depends on a new TAL effector that induces the rice nodulin-3 Os11N3 gene. *Molecular plant-microbe interactions : MPMI*, **24**, 1102–13.

3. Wonni,I., Cottyn,B., Detemmerman,L., Dao,S., Ouedraogo,L., Sarra,S., Tekete,C., Poussier,S., Corral,R., Triplett,L., *et al.* (2014) Analysis of Xanthomonas oryzae pv. oryzicola Population in Mali and Burkina Faso Reveals a High Level of Genetic and Pathogenic Diversity. *Phytopathology*, **104**, 520–31.

4. Wilkins,K.E., Booher,N.J., Wang,L. and Bogdanove,A.J. (2015) TAL effectors and activation of predicted host targets distinguish Asian from African strains of the rice pathogen Xanthomonas oryzae pv. oryzicola while strict conservation suggests universal importance of five TAL effectors. *Frontiers in Plant Science*, **6**, 536.

5. Bogdanove,A.J., Koebnik,R., Lu,H., Furutani,A., Angiuoli,S.V., Patil,P.B., Van Sluys,M.-A., Ryan,R.P., Meyer,D.F., Han,S.-W., *et al.* (2011) Two new complete genome sequences offer insight into host and tissue specificity of plant pathogenic Xanthomonas spp. *J Bacteriol*, **193**, 5450–5464.

6. Makino,S., Sugio,A., White,F. and Bogdanove,A.J. (2006) Inhibition of resistance gene-mediated defense in rice by Xanthomonas oryzae pv. oryzicola. *Mol Plant Microbe Interact*, **19**, 240–249.

7. Zhao,S., Poulin,L., Rodriguez-R,L.M., Serna,N.F., Liu,S.-Y., Wonni,I., Szurek,B., Verdier,V., Leach,J.E., He,Y.-Q., *et al.* (2012) Development of a variable number of tandem repeats typing scheme for the bacterial rice pathogen Xanthomonas oryzae pv. oryzicola. *Phytopathology*, **102**, 948–956.

8. Lee,B.-M., Park,Y.-J., Park,D.-S., Kang,H.-W., Kim,J.-G., Song,E.-S., Park,I.-C., Yoon,U.-H., Hahn,J.-H., Koo,B.-S., *et al.* (2005) The genome sequence of Xanthomonas oryzae pathovar oryzae KACC10331, the bacterial blight pathogen of rice. *Nucleic Acids Res*, **33**, 577–586.

9. Moscou,M.J. and Bogdanove,A.J. (2009) A simple cipher governs DNA recognition by TAL effectors. *Science (New York, N.Y.)*, **326**, 1501.

10. Verdier,V., Triplett,L.R., Hummel,A.W., Corral,R., Cernadas,R.A., Schmidt,C.L., Bogdanove,A.J. and Leach,J.E. (2012) Transcription activator-like (TAL) effectors targeting OsSWEET genes enhance virulence on diverse rice (Oryza sativa) varieties when expressed individually in a TAL effector-deficient strain of Xanthomonas oryzae. *The New phytologist*, **196**, 1197–207.

11. Gonzalez,C., Szurek,B., Manceau,C., Mathieu,T., Séré,Y. and Verdier,V. (2007) Molecular and pathotypic characterization of new Xanthomonas oryzae strains from West Africa. *Molecular plant-microbe interactions : MPMI*, **20**, 534–46.

12. Hopkins,C.M., White,F.F., Choi,S.H., Guo,A., Leach,J.E. and C.M. Hopkins, F. F. White, S.-H. Choi, A. Guo,J.E.L. (1992) Identification of a family of avirulence genes from xanthomonas oryzae pv. oryzae. *Molecular Plant Microbe Interactions*, **5**, 451–459.

13. Zhu,W., MaGbanua,M.M. and White,F.F. (2000) Identification of two novel hrp-associated genes in the hrp gene cluster of Xanthomonas oryzae pv. oryzae. *J Bacteriol*, **182**, 1844–1853.

14. Triplett,L.R., Hamilton,J.P., Buell,C.R., Tisserat,N.A., Verdier,V., Zink,F. and Leach,J.E. (2011) Genomic analysis of Xanthomonas oryzae isolates from rice grown in the United States reveals substantial divergence from known X. oryzae pathovars. *Appl Environ Microbiol*, **77**, 3930–3937.

15. Blanvillain-Baufumé,S., Reschke,M., Solé,M., Auguy,F., Doucoure,H., Szurek,B., Meynard,D., Portefaix,M., Cunnac,S., Guiderdoni,E., *et al.* (2017) Targeted promoter editing for rice resistance to Xanthomonas oryzae pv. oryzae reveals differential activities for SWEET14-inducing TAL effectors. *Plant Biotechnology Journal*, **15**.

16. Liu,B., Chen,Z., Song,X., Liu,C., Cui,X., Zhao,X., Fang,J., Xu,W., Zhang,H., Wang,X., *et al.* (2007) Oryza sativa dicer-like4 reveals a key role for small interfering RNA silencing in plant development. *Plant Cell*, **19**, 2705–2718.

17. Abe,M., Yoshikawa,T., Nosaka,M., Sakakibara,H., Sato,Y., Nagato,Y. and Itoh,J. (2010) WAVY LEAF1, an ortholog of Arabidopsis HEN1, regulates shoot development by maintaining MicroRNA and trans-acting small interfering RNA accumulation in rice. *Plant Physiol.*, **154**, 1335–1346.
